# Salmonid Chromosome Evolution as Revealed by a Novel Method for Comparing RADseq Linkage Maps

**DOI:** 10.1093/gbe/evw262

**Published:** 2016-11-09

**Authors:** Ben J. G. Sutherland, Thierry Gosselin, Eric Normandeau, Manuel Lamothe, Nathalie Isabel, Céline Audet, Louis Bernatchez

**Affiliations:** 1 Institut de Biologie Intégrative et des Systèmes (IBIS), Université Laval, Québec, QC, Canada; 2 Centre de Foresterie des Laurentides, Ressources Naturelles Canada, Québec, QC, Canada; 3 Institut des Sciences de la Mer de Rimouski, Université du Québec à Rimouski, Rimouski, QC, Canada

**Keywords:** comparative genomics, linkage map, RADseq, salmon, whole genome duplication

## Abstract

Whole genome duplication (WGD) can provide material for evolutionary innovation. Family Salmonidae is ideal for studying the effects of WGD as the ancestral salmonid underwent WGD relatively recently, ∼65 Ma, then rediploidized and diversified. Extensive synteny between homologous chromosome arms occurs in extant salmonids, but each species has both conserved and unique chromosome arm fusions and fissions. Assembly of large, outbred eukaryotic genomes can be difficult, but structural rearrangements within such taxa can be investigated using linkage maps. RAD sequencing provides unprecedented ability to generate high-density linkage maps for nonmodel species, but can result in low numbers of homologous markers between species due to phylogenetic distance or differences in library preparation. Here, we generate a high-density linkage map (3,826 markers) for the *Salvelinus* genera (Brook Charr *S. fontinalis*), and then identify corresponding chromosome arms among the other available salmonid high-density linkage maps, including six species of *Oncorhynchus*, and one species for each of *Salmo*, *Coregonus*, and the nonduplicated sister group for the salmonids, Northern Pike *Esox lucius* for identifying post-duplicated homeologs. To facilitate this process, we developed MapComp to identify identical and proximate (i.e. nearby) markers between linkage maps using a reference genome of a related species as an intermediate, increasing the number of comparable markers between linkage maps by 5-fold. This enabled a characterization of the most likely history of retained chromosomal rearrangements post-WGD, and several conserved chromosomal inversions. Analyses of RADseq-based linkage maps from other taxa will also benefit from MapComp, available at: https://github.com/enormandeau/mapcomp/

## Introduction

Whole genome duplication (WGD) can provide the raw material for evolutionary innovation by generating copies of all chromosomes (i.e. producing homeologous chromosome pairs). After WGD, the genome can then undergo rediploidization while retaining all duplicated chromosome arms (homeologs), thereby doubling the pre-duplication chromosome arm number. Rediploidization remains a highly studied topic and probably involves large-scale structural changes between the homeologous chromosomes such as massive repeat element expansion (Lien et al. 2016). Further divergence between homeologous chromosomes occurs at the level of the gene, where gene copies can evolve new functions, sub-functionalize the original function between the two copies or, most frequently, accumulate mutations that disrupt functionality of one copy (Ohno 1970; Force et al. 1999; Brunet et al. 2006). Cross-taxa analyses suggest that pseudogenization often results in singletons being retained preferentially on one of the two homeologs (Sankoff et al. 2010), but nonrandom retention is not always observed (Berthelot et al. 2014). Interestingly, rediploidization does not always complete. For example, in salmonids some homeologous chromosome arms continue recombining, resulting in residual tetraploidy (Allendorf et al. 2015).

Eukaryotic genomes with ancestral WGD and residual tetraploidy are challenging to assemble (Davidson et al. 2010). Linkage maps can be highly useful for comparing chromosomal evolution among lineages using shared markers between maps to identify corresponding chromosomes (i.e. homologous chromosomes) between species (Naish et al. 2013; Kodama et al. 2014). Furthermore, high quality, dense linkage maps are valuable for validating and orienting genomic scaffolds (Mascher & Stein 2014; Fierst 2015), especially for cases of residual polyploidy, large genome size, and high repeat content (Amores et al. 2014; Ming & Man Wai 2015). Recent advances in sequencing, such as through reduced-representation library sequencing (e.g. RADseq) (Baird et al. 2008; Elshire et al. 2011; Andrews et al. 2016), have made high-density linkage maps increasingly easy to produce. These methods provide thousands of markers without requiring marker design effort (Catchen et al. 2011). RADseq-based SNP markers are contained in short sequence fragments, which allow for mapping against a genome to identify nearby genes or physical distances between markers (Amores et al. 2011; Henning et al. 2014). RADseq also enables comparative genomics through the use of direct marker-to-marker comparisons to find homologous markers between linkage maps (Kodama et al. 2014). The ability to identify homologous chromosomes between maps is dependent on being able to identify enough shared markers between species, and this decreases with phylogenetic distance due to sequence divergence (Gonen et al. 2015). This issue is compounded further when different protocols or restriction enzymes are used for library generation. Due to this, it has been suggested to use a common enzyme and protocol to ensure compatibility of maps (Larson et al. 2016) to provide markers shared between species similar to shared microsatellite markers (Danzmann et al. 2005), but this is not always performed. Here we developed a method to use a related species’ reference genome to integrate linkage maps of different species by pairing homologous and proximate (i.e. nearby) markers from pairs of species. We demonstrate the utility of this method in the salmonids, and expand the comparative genomics of this taxon to provide the most comprehensive analysis to date of the post-WGD chromosome evolution of the salmonids in terms of chromosome arm fusions, fissions and large-scale inversions.

Salmonids are a useful study system for investigating the effects of WGD. The ancestor of modern day salmonids, probably having a karyotype similar to the extant Northern Pike *Esox lucius* (which is a member of Esocidae, the sister family of Salmonidae) with 25 acrocentric chromosomes (Rondeau et al. 2014), underwent WGD ∼65 Ma, and subsequently underwent rediploidization (Allendorf & Thorgaard 1984; Davidson et al. 2010). Post-WGD, the salmonid lineage diversified into three subfamilies, 11 genera and more than 60 described species (Crête-Lafrenière et al. 2012), although this diversification was likely due to environmental factors rather than being caused by WGD (Macqueen & Johnston 2014). Analysis of the Atlantic Salmon genome suggests that the rediploidization process was rapid and that two classes of homeolog similarity exist: immediately rediploidized homeologs and those in residual tetraploidy that continue to recombine between homeologs (see Figure 3b in Lien et al. 2016). Although much remains to be understood about rediploidization and residual tetraploidy in salmonids, fundamental work on chromosomal evolution has been conducted using cytogenetics and genetic maps (Phillips & Ráb 2001; Naish et al. 2013). From linkage map comparisons using homologous markers, it is known that the same eight pairs of corresponding homeologs are residually tetraploid in Chinook Salmon (Brieuc et al. 2014), Coho Salmon (Kodama et al. 2014) and Sockeye Salmon (Larson et al. 2016), as well as some in Atlantic Salmon although some of these have lower support (Lien et al. 2011)*.* The consistency of these residually tetraploid homeologs indicates that prevention of rediploidization in these chromosomes occurred prior to the divergence of these species (Kodama et al. 2014).

Chromosomal evolution within family Salmonidae (i.e. whitefish, trout, charr and salmon) is typified by centric Robertsonian fusions (hereafter metacentric fusions), whereby two acrocentric chromosomes fuse into one larger metacentric chromosome, retaining the total number of chromosome arms (*nombre fondamental* (NF) = 100) but differing in total chromosome number (Phillips & Ráb 2001). Fissions and whole arm translocations can also occur, subsequently separating the fused metacentric chromosomes. Cytogenetic research has identified the presence of two major karyotype groups in salmonids differing in the number of retained chromosome fusion events. Type A species (2*n* = ∼80 chromosomes) have more acrocentric than metacentric chromosomes, whereas Type B species (2*n* = ∼60 chromosomes) have more metacentric than acrocentric chromosomes (Phillips & Ráb 2001). Adaptive mechanisms or selective forces driving these rearrangements and correlation with habitat or species biology remain generally unknown (Phillips & Ráb 2001).

In general, retained collinearity is expected between homologous chromosome arms among species (Kodama et al. 2014) and between homeologous chromosomes within a species (Berthelot et al. 2014). Using comparative mapping with homologous markers, the conservation and timing of chromosome fusions has been described between two Pacific salmon species, Chinook *O. tshawytscha* and Coho Salmon *O. kisutch*, and an Atlantic salmonid, Atlantic Salmon *Salmo salar* (Kodama et al. 2014). This work provided evidence that at least one of the homeologs exhibiting residual tetraploidy was fused in a metacentric chromosome prior to the divergence of *Salmo* and *Oncorhynchus*. This comparative analysis has not yet been extended across other genera with genetic maps available, including the genus *Coregonus* (more basal than *Salmo*), and genera without high-density maps available (e.g. *Salvelinus*), and this increased taxonomic sampling would provide new insights on the timing and process of chromosome arm fusion and fission post-WGD. Considering the important role of metacentric fusions in the rediploidization process, probably due to a higher frequency of tetravalents occuring at meiosis (Wright et al. 1983; Phillips et al. 2009; Brieuc et al. 2014; Kodama et al. 2014; Allendorf et al. 2015; May & Delany 2015), the investigation of these fusions is crucial to understand rediploidization in this taxon.

High-density linkage maps have been constructed for Lake Whitefish *Coregonus clupeaformis* (Gagnaire et al. 2013), Atlantic Salmon *S. salar* (Lien et al. 2011; Gonen et al. 2014) and members of *Oncorhynchus* including Rainbow Trout *O. mykiss* (Miller et al. 2012; Palti et al. 2015), Chinook Salmon *O. tshawytscha* (Brieuc et al. 2014), Coho salmon *O. kisutch* (Kodama et al. 2014), Pink Salmon *O. gorbuscha* (Limborg et al. 2014), Chum Salmon *O. keta* (Waples et al. 2016) and Sockeye Salmon *O. nerka* (Everett et al. 2012; Larson et al. 2016). No high-density maps exist for members of *Salvelinus*, but low-density microsatellite-based maps exist for Arctic Charr *S. alpinus* and Brook Charr *S. fontinalis* (Woram et al. 2004; Timusk et al. 2011), as well as a low-density (∼300 marker) EST-derived SNP map for *S. fontinalis* (Sauvage et al. 2012a). Genome assemblies exist for Rainbow Trout (Berthelot et al. 2014) and Atlantic Salmon (Lien et al. 2016). A genome assembly and low-density genetic map are also available for Northern Pike *Esox lucius*, a sister species to the salmonid WGD (Rondeau et al. 2014). With these resources available, it becomes especially valuable to integrate the information from all of the maps to detail the chromosomal evolution of the salmonids.

In this study, we use a mapping family previously used to generate a low-density EST-derived SNP linkage map (Sauvage et al. 2012a) to produce the first high-density RADseq map for the genus *Salvelinus*, the Brook Charr *S. fontinalis*. Brook Charr is a species of importance for conservation, aquaculture and fisheries, and an underrepresented lineage of Salmonidae in terms of genomic resource availability. To facilitate and automate the identification of homologous and homeologous chromosomes within the available salmonid resources, we developed MapComp, a program to compare genetic maps built from related species with or without the same RADseq protocol using a reference genome of a related species as an intermediate. MapComp follows earlier proposed approaches to integrate nonmodel maps with model species genomes (Sarropoulou et al. 2008). It identifies on an average 5-fold more marker pairs between linkage maps than methods relying on homologous markers only, and creates pairwise comparison plots for data visualization. MapComp enabled a detailed characterization of the homologous and homeologous chromosome arms representing all if the main genera comprised within the salmonid family, and thus the characterization of the most likely historical chromosomal rearrangements occurring at different levels of the salmonid phylogeny, including some potential inversion events. This comprehensive view provides new insight on the post-WGD chromosome evolution of Family Salmonidae.

## Materials and Methods

### Brook Charr Genetic Map

#### Animals

Full details regarding the experimental mapping family were reported previously (Sauvage et al. 2012a, 2012b). The F_0_ female was from a wild anadromous population from Laval River (near Forestville, Québec) that have been kept in captivity for three generations at the Station aquicole de l’ISMER (Rimouski, Québec), and the F_0_ male was from a domestic population used in Québec aquaculture for 100 years, supplied here from the Pisciculture de la Jacques–Cartier (Cap-Santé, Québec). Three biparental crosses of F_1_ individuals produced three F_2_ families, and the family with the largest number of surviving offspring was chosen to be the mapping family (*n* = 192 full-sib F_2_ offspring).

#### DNA Extraction, Sample Preparation and Sequencing

DNA was extracted from the fin of F_2_ offspring and F_1_ parents by high salt extraction (Aljanabi & Martinez 1997) with an additional RNase A digestion step (QIAGEN), as previously reported (Sauvage et al. 2012a). Quality of the extracted genomic DNA was quality validated by gel electrophoresis and quantified using Quant-iT PicoGreen double-stranded DNA Assay (Life Technologies) using a Fluoroskan Ascent FL fluorometer (Thermo LabSystems).

Double-digest RADseq (Baird et al. 2008) was performed as per methods previously outlined (Elshire et al. 2011) and described in full elsewhere (Poland et al. 2012). Briefly, two restriction enzymes were used (*Pst*I and *Msp*I) to digest genomic DNA. Digested DNA was then ligated with adapters and barcodes for individual identification then amplified by PCR. For the offspring, uniquely barcoded individuals were then combined in equimolar proportions into eight pools, each pool containing 25 individuals. Pools were each sequenced on a single lane on a HiSeq2000 at Génome Québec Innovation Centre (McGill University, Montréal). In order to obtain deeper sequencing of the parents, each parent individual was sequenced using an Ion Torrent at the sequencing platform at IBIS (Institut de Biologie Intégrative et des Systèmes, Université Laval, Québec City). This platform change between F_1_ and F_2_ individuals occurred due to equipment availability, but extra precaution was taken to ensure proper correspondence of loci (see below).

#### Bioinformatic Pipeline and Reduced Genome De Novo Assembly

Raw reads were inspected for overall quality and presence of adapters with fastqc (http://www.bioinformatics.babraham.ac.uk/projects/fastqc/; last accessed November 8, 2016). Adapters were removed and raw reads were truncated to 80 bp using cutadapt v.1.9.dev.0 (Martin 2011). Reads were de-multiplexed by barcodes, and quality trimmed to 80 bp using the stacks v.1.32 (Catchen et al. 2011, 2013) *process_radtags* module. The ploidy-informed empirical procedure was used (Ilut et al. 2014) to optimize *de novo* assembly. Sequence similarity was explored to find the optimum clustering threshold, which is highly important for salmonid *de novo* assembly due to residual tetraploidy (see supplementary file S1 for pipeline parameters, Supplementary Material online). Data from each individual were grouped into loci, and polymorphic nucleotide sites were identified with the *ustacks* module. The catalog construction used all loci identified across the parents. Differentially fixed loci (i.e. monomorphic loci among parents) were allowed to merge as a single locus when no mismatches were found (*cstacks*). Loci from parents and offspring were matched against the parental catalog to determine the allelic state at each locus in each individual in *sstacks*. To improve the quality of the *de novo* assemblies produced in stacks and to reduce the risk of generating problematic loci with repetitive sequences and paralogs, we used the correction module *rxstacks*. A haploid cross is required to fully investigate paralogous loci (Kodama et al. 2014), and so here we removed these loci. The log-likelihood threshold for *rxstacks* was chosen based on the distribution of mean and median log-likelihood values. After the correction module, the catalog and individuals’ matches were rebuilt with the corrected individuals files. The *genotypes* module of stacks was used to output markers along with their allelic state and raw genotypes. The markers were translated using the function *genotypes_summary.R* of stackr (Gosselin & Bernatchez) into fully informative (i.e. informative for both parents) or semi-informative (i.e. partially informative or informative in only one parent) marker types, specifically the four types of markers that permitted in the outbreeding design: *ab*×*ac*, *ab*×*ab*, *ab*×*aa* and *aa*×*ab* (Wu et al. 2002).

#### Pre-Mapping Quality Control

Several steps of quality control were performed based on recommendations (van Ooijen & Jansen 2013). Pre-mapping quality control consisted of excluding individuals with > 30% missing data (22 progeny), monomorphic loci and loci with an incomplete segregation pattern inferred from the parents (i.e. missing alleles) using the *genotypes_summary.R* function. This function was also used to filter errors in the phenotype observations of markers with a segregation distortion filter using a chi-squared goodness-of-fit test (*filter.GOF*). With heterozygous parents, not all of the markers contribute equally to the construction of the map, because linkage phases change across loci (van Ooijen & Jansen 2013). Therefore, tolerance for genotyping errors (goodness-of-fit threshold: 12–20) and missing genotypes (50–90% thresholds) were also explored with *genotypes_summary.R*. Different thresholds among the marker types were used to maximally retain the informative markers and to increase stringency on the less informative *ab*×*ab* markers.

#### Linkage Mapping and Post-Mapping Quality Control

The linkage map was first built in JoinMap v4.1 (van Ooijen 2006) using the pseudo-testcross approach (Grattapaglia & Sederoff 1994; van Ooijen & Jansen 2013) that only uses the markers segregating in a uni-parental configuration (i.e. *ab*×*ac* and *ab*×*ab* markers are excluded here). Subsequently, additional maps were produced (consensus, male and female) using all markers in an analyses using a CP population type (cross pollinator, or full-sib family) with the multipoint maximum likelihood mapping algorithm for marker order (van Ooijen 2011; van Ooijen & Jansen 2013). The initial pseudo-testcross maps were used for confirmation of the multipoint maximum likelihood maps. Separate maximum likelihood maps were generated for each parent, and only the female map was retained, as is typical for salmonid mapping studies (Kodama et al. 2014) due to low recombination rate observed in male salmonids (Sakamoto et al. 2000). Markers were grouped with the independent LOD option of JoinMap with a range of 15–40 LOD, for the minimum and maximum threshold, respectively. A total of 42 linkage groups (LGs) were defined by evaluating stability of marker numbers over increasing consecutive LOD values. This number of LGs corresponds to the expected chromosome number of Brook Charr (2*n* = 84). During mapping, the stabilization criterion was monitored in the session log with the sum of recombination frequencies of adjacent segments and the mean number of recombination events. Default mapping parameters usually performed well with the smaller LG, but for larger LG the stabilization was not always reached, so more EM cycles and longer chains per cycle were used. For full details of parameters used in JoinMap, see supplementary file S1, Supplementary Material online.

Problematic or unlinked markers and small linkage groups were tested using several JoinMap features (i.e. *crosslink*, *genotype probabilities*, *fit* and *stress*) as recommended by van Ooijen and Jansen (2013) to detect errors in ordering and genotyping for excluding markers with these criteria: (1) oversized LG, which can occur with high marker numbers; (2) incidence of improbable genotypes e.g. double recombinants (Henning et al. 2014); (3) drastic changes of order after single markers were removed; and (4) low levels of fit or high levels of stress. Maps were inspected for distortion before and after manual exclusion of markers. Mapping distances (cM) were calculated using the Haldane mapping function.

### MapComp

#### Map Comparison through Intermediate Reference Genome

In order to compare the Brook Charr map to other salmonid maps, published linkage map datasets were collected (see the “Code and Pipeline Availability” section), including information on marker name, sequence, linkage group and cM position. Comparisons of linkage group correspondence and synteny between species were investigated using available high-density linkage maps. This included maps generated with haploid crosses for mapping regions exhibiting residual tetraploidy (Limborg et al. 2016), although in most cases, we only retained the nonduplicated loci due to problems in pairing these markers (described in workflow below). Some of these maps also contain centromere information, including Chinook Salmon (Brieuc et al. 2014), Coho Salmon (Kodama et al. 2014), Sockeye Salmon (Everett et al. 2012; Limborg et al. 2015), and Chum Salmon (Waples et al. 2016). A high-density map for Atlantic Salmon with information on duplicate regions is also available (Lien et al. 2011). Other available high-density maps from diploid crosses included Pink Salmon (Limborg et al. 2014), Rainbow Trout (Miller et al. 2012; Palti et al. 2015), Lake Whitefish (Gagnaire et al. 2013), and the salmonid WGD sister outgroup Northern Pike (Rondeau et al. 2014) (see table 1).

**Table 1:** Overview of Compared Species

Common Names with References	Genus and Species Names	Map Type (Num. Markers)	Number of Chr. (1n)	**Exp. Genome Size (*C* value)** (Gregory 2016)	Exp. Genome Size (Gbp)
Northern Pike^a^ (Rondeau et al. 2014)	*Esox lucius*	EST-based microsatellite (524)	25	0.85–1.40	0.8–1.4
Lake Whitefish (Gagnaire et al. 2013)	*Coregonus clupeaformis*	RADseq with *Sbf*I (3,438)	40	2.44–3.44	2.4–3.4
Atlantic Salmon (Lien et al. 2011)	*Salmo salar*	EST-based SNP chip (5,650)	29	2.98–3.27	2.8–3.2
Brook Charr	*Salvelinus fontinalis*	RADseq with *Pst*I and *Msp*I (3,826)	42	2.86–3.50	2.8–3.4
Rainbow Trout (Palti et al. 2015)	*Oncorhynchus mykiss*	RADseq with *Sbf*I (955)	29	1.87–2.92	1.8–2.9
Coho Salmon (Kodama et al. 2014)	*O. kisutch*	RADseq with *Sbf*I (5,377)	30	2.60–3.05	2.5–3.0
Chinook Salmon (Brieuc et al. 2014)	*O. tshawytscha*	RADseq with *Sbf*I (6,352)	34	2.45–3.30	2.4–3.2
Pink Salmon (Limborg et al. 2014)	*O. gorbuscha*	RADseq with *Sbf*I (7,035)	26	2.23–2.57	2.2–2.5
Chum Salmon (Waples et al. 2016)	*O. keta*	RADseq with *Sbf*I (6,119)	37	2.49–2.76	2.4–2.7
Sockeye Salmon (Larson et al. 2016)	*O. nerka*	RADseq with *Sbf*I (6,262)	29	2.77–3.04	2.7–3.0

Note.—The common and scientific name for each species in the analysis are displayed along with the source of the genetic map, the type of map and number of markers, the chromosome number for the species, and expected genome size (*C* value and Gbp) obtained from (Gregory 2016). The number of chromosome arms for each species is 50, although this may be polymorphic in some species. The number of chromosomes is known to be 28 for males and 29 for females in Sockeye Salmon; here and in table 2, we list the second half of LG09 as LG29. The order of species here starts with the nonduplicated sister group Northern Pike followed by the more ancestrally diverging taxa to the recently diverged (*Oncorhynchus*), and this order is retained throughout.

a Sister species to salmonid WGD.

**Fig. 1.—:**
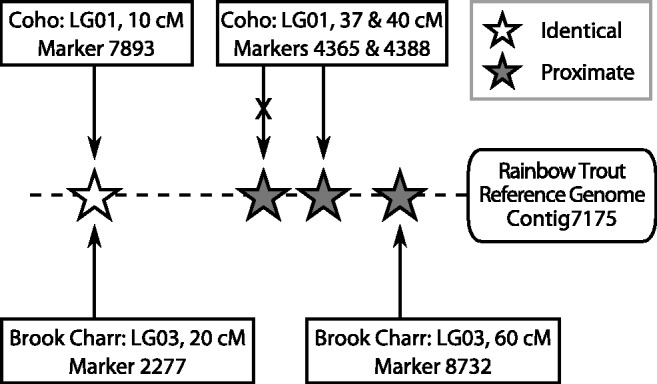
Schematic of MapComp using a reference genome to pair markers. MapComp compares genetic maps from two different species (e.g. Coho Salmon and Brook Charr) by mapping marker sequences against a reference genome (e.g. Rainbow Trout), then retaining high quality mappings (i.e. single alignment with MAPQ ≥10). The closest two markers, one from each species, are paired if they align to the same contig/scaffold. This method captures homologous markers (e.g. the white star in image) and nonidentical but nearby markers (e.g. gray stars). In this example, the closest marker from Coho Salmon and Brook Charr are paired, and the second closest marker from Coho Salmon is discarded (shown with an X) because each marker is paired without replacement. After pairing is complete, the cM position of the marker from each species’ linkage group is plotted in an Oxford grid. The position on the contig is not carried over to the Oxford grid, only that from the linkage maps. Note that the marker names and contig ID in the schematic are for demonstration purposes only and do not reflect actual pairings.

The basic workflow of MapComp is shown in figure 1. First, all marker sequences were combined into a single fasta file and mapped to a reference genome, here the Rainbow Trout scaffolds (http://www.genoscope.cns.fr/trout/data/; last accessed November 8, 2016) (Berthelot et al. 2014) or the Atlantic Salmon assembly ICSASG_v2 (Lien et al. 2016) using *BWA mem* (Li & Durbin 2009). Using the basic workflow of MapComp, matches to the reference genome are only retained when a single alignment occurs with a MAPQ score ≥ 10. It is important to note that even though some of the regions of the salmonid genome exhibits residual tetraploidy, these regions are probably collapsed in the genome assembly and so will not be present in duplicate in the assembly (Lien et al. 2016). However, MapComp may not be able to resolve which of the duplicated markers to pair in these regions, and so for the purpose of identifying homologous chromosome arms, clearer results are obtained when it is possible to exclude duplicate markers in the maps to be compared. After aligning the genetic maps of both species to the genome assembly, the pairs of markers (one from each species) that are closest to each other in position on the same contig or scaffold are paired. Pairing occurs without replacement (i.e. once the closest marker pair was selected, other markers also pairing with the marker that has now been paired were then discarded). Each marker pair is then added to an Oxford grid. The pipeline developed for MapComp is available at https://github.com/enormandeau/mapcomp/.

To identify homeologous relationships using MapComp, where two chromosome arms originate from the same pre-duplicated chromosome arm, comparisons between Northern Pike *E. lucius* and the other salmonids were conducted. This required changing some parameters in MapComp to allow for multiple hits from the nonduplicated Northern Pike map against the Atlantic Salmon and Rainbow Trout reference genome intermediates, as each marker could be present in at least duplicate in the salmonid genome. Specifically, the mapping quality threshold was lowered (MAPQ ≥ 2) and mapping against more than one locus in the Rainbow Trout or Atlantic Salmon reference genome was permitted.

#### Characterization of Homology and Homeology between Chromosome Arms

Homology of chromosome arms between Chinook Salmon and Coho Salmon maps identified previously using homologous markers (Kodama et al. 2014) was confirmed using MapComp. Chinook Salmon and Coho Salmon were then individually compared with Brook Charr to identify corresponding chromosome arms in Brook Charr. Once these homologous relationships were obtained, the Brook Charr map was compared with Pink Salmon, Sockeye Salmon, Chum Salmon, Rainbow Trout and Atlantic Salmon genetic maps. Chromosome correspondence was identified in Lake Whitefish using a consensus approach, where results from comparisons of Lake Whitefish with multiple different species were considered for unambiguous determination of homology. Homeologs were also identified using a consensus approach, and the original Northern Pike linkage groups were given .1 or .2 designations to represent the duplicated chromosomes. These results were compared with the results of Rondeau et al. (2014), in which blast was used with Atlantic Salmon linkage groups against the Northern Pike genome to identify salmonid WGD homeologs.

#### Identification of Putative Inversions

Plots from MapComp were visually inspected for inversions. During linkage mapping, when markers do not fit in the linkage group, they might be placed at the distal ends of the linkage group (Henning et al. 2014). Therefore, to avoid the erroneous identification of inversions, evidence for inversions was only considered when noninverted regions flanked the inverted region. As the analysis is based on linkage maps and not assembled genomes, all inversions were considered putative. Furthermore, phylogenetic relationships and inversion conservation across species were considered (i.e. when an inversion was identified within multiple species within a lineage). Centromere locations were obtained from Chinook Salmon (Brieuc et al. 2014) to allow the characterization of selected inversions as either pericentric (involving the centromere) or paracentric (not involving the centromere).

#### Conservation of Rearrangements and Identification of Full Coverage of Linkage Groups

The conservation of chromosomal rearrangements among the salmonids was analyzed by using the most taxonomically complete phylogeny of the salmonids (Crête-Lafrenière et al. 2012) but with Rainbow Trout as the outgroup to the other *Oncorhynchus* clades as reported previously (Kinnison & Hendry 2004), and with the still-debated clade containing Pink Salmon, Chum Salmon and Sockeye Salmon arranged in the most parsimonious phylogeny in terms of the numbers of required fusions/fissions (see table 1). The analysis of metacentric conservation was based on the analysis of conservation in Coho, Chinook, Rainbow Trout and Atlantic Salmon (Kodama et al. 2014), but re-analyzed using MapComp and additional maps in the present study (i.e. Pink Salmon, Chum Salmon, Sockeye Salmon, Brook Charr and Lake Whitefish). To confirm that a metacentric chromosome was completely present, for conserved metacentric identification, we required evidence from both sides of the centromere.

## Results

### Generation of a Brook Charr Linkage Map

On an average, 10M single-end reads were obtained for each parent and 5M for each individual offspring. Using stacks v1.32 (Catchen et al. 2011), 6,264 segregating markers were identified, each containing one to five SNPs within the same read. Missing data per marker followed a heavy-tailed distribution, having a mode of 10 individuals genotypes missing for ∼700 markers. Female, male and consensus genetic maps were generated, but the female-specific map (*n* = 3,826 markers) was retained as the final map, as is typical for salmonids due to low recombination rate in males (Brieuc et al. 2014; Kodama et al. 2014).

**Fig. 2.—:**
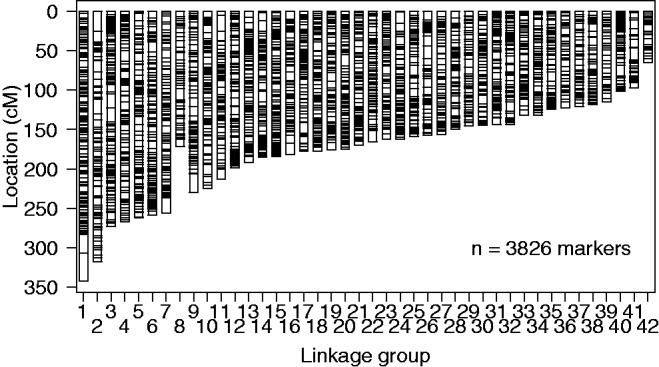
Brook Charr *Salvelinus fontinalis* linkage map. Eight metacentric (LG1–8) and 34 acrocentric linkage groups (LG9–42) were identified in the female map. Metacentric chromosomes were identified through homologous relationships of chromosome arms with other salmonids. Horizontal lines within each linkage group are markers (total = 3826 markers).

A total of 42 linkage groups were characterized in the female map (fig. 2), corresponding to the expected haploid chromosome number for Brook Charr (Phillips & Ráb 2001). On average, metacentric linkage groups were 270 cM (range = 185–342 cM) containing 126 markers (range = 107–175 markers), whereas acrocentric linkage groups were 156 cM (range = 65–230 cM) containing 83 markers (range = 33–134). The total length of the female map was 7,453.9 cM. Descriptive statistics for the linkage groups are in supplementary file S2, Supplementary Material online. This size is in the range of other high-density salmonid maps, such as the Coho Salmon linkage map (6,596.7 cM) (Kodama et al. 2014), although is larger than the Chinook Salmon map (4,164 cM) (Brieuc et al. 2014). The female map contains 3,826 markers with the following marker types, as defined by Wu et al. (2002): 254 fully informative (*ab*×*ac*), 954 semi-informative (*ab*×*ab*) and 2,618 fully informative in female parent (*ab*×*aa*). The female map is in supplementary file S3, Supplementary Material online. The consensus map contained an additional 2,385 markers that were informative in the male parent (*aa*×*ab*) but although these markers were in the correct linkage groups, they did not position well within the linkage group. This is most likely due to the low recombination rate known to occur in male salmonids, as almost complete crossover interference can occur within male salmonids during meiosis (Naish et al. 2013).

**Fig. 3.—:**
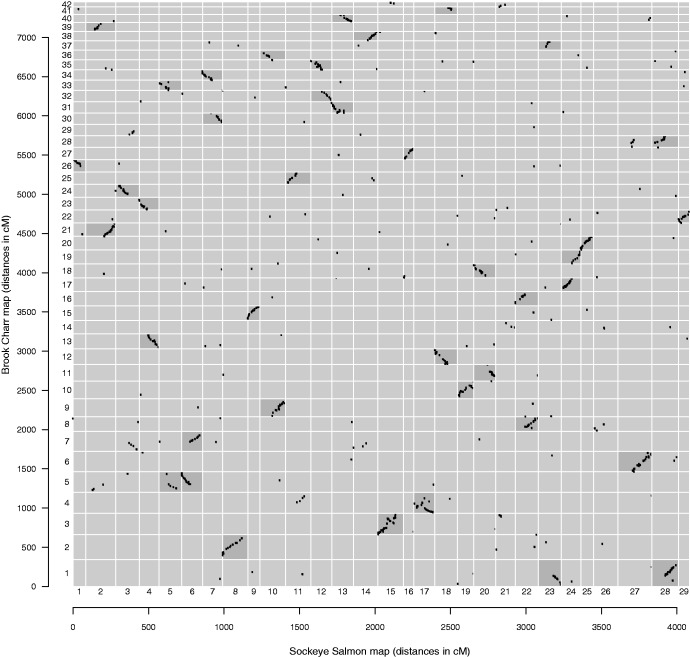
MapComp determination of homologous chromosome arms. Brook Charr compared with Sockeye Salmon with markers paired through the Rainbow Trout genome identifies homology between chromosome arms. A putative inversion can be seen between Brook Charr LG03 and Sockeye Salmon LG15.

### Identification of Homologous Chromosome Arms among the Salmonids

Assignment of linkage groups to chromosome arms has been performed using fluorescence in situ hybridization with BAC probes for Atlantic Salmon (Phillips et al. 2009) and Rainbow Trout (Phillips et al. 2006), and synteny has been designated using homologous microsatellite and RADseq markers (using the same library preparation protocols) among Chinook Salmon, Coho Salmon, Rainbow Trout and Atlantic Salmon (Danzmann et al. 2008; Phillips et al. 2009; Naish et al. 2013; Brieuc et al. 2014; Kodama et al. 2014) and recently Sockeye Salmon (Larson et al. 2016). A full comparison across all existing maps has yet to be completed. The low-density linkage map of the Northern Pike *E. lucius* has been compared with Atlantic Salmon (Rondeau et al. 2014), but not yet with the rest of the salmonids. Details on the linkage maps and species used in this analysis, including expected genome sizes (Gregory 2016) are provided in table 1. It is important to note that the linkage group names and orientations within this analysis were obtained from these datasets.

To begin homologous designation of linkage groups, Chinook Salmon and Coho Salmon linkage maps were used to compare with the map of Brook Charr using MapComp pairing markers through the Rainbow Trout genome sequence (Berthelot et al. 2014) (see MapComp schematic in fig. 1, and Methods for full details). All chromosome arms (NF = 50) were identified unambiguously in Brook Charr (fig. 3 and table 2). The Brook Charr linkage map was then compared with linkage maps of Sockeye Salmon, Chum Salmon, Pink Salmon, Rainbow Trout and Atlantic Salmon (table 2). In a few rare cases where homology with Brook Charr was not obvious, species were also compared with Chinook Salmon or others to clearly indicate the corresponding chromosome arm. One chromosome arm in Rainbow Trout required the use of a second Rainbow Trout linkage map to unambiguously identify homologous arms (Miller et al. 2012). Most arms were also identified in the more distantly related Lake Whitefish, but seven arms remained ambiguous or unidentifiable.

**Table 2:** Homologous Chromosome Arms across the Salmonids

Northern Pike	Lake Whitefish	Atlantic Salmon	Brook Charr	Rainbow Trout	Coho Salmon	Chinook Salmon	Pink Salmon	Chum Salmon	Sockeye Salmon
1.1	Cclu28	**Ssa20b**	BC25	Omy27	Co15b	Ots13q	Og13b	Ok18	On11a
1.2	Cclu35	**Ssa09c**	BC38	Omy24	Co18a	Ots14q	Og19b	Ok01a	On14b
2.1	*Cclu04a?*	**Ssa26**	BC06a	Omy06b	**Co03b**	**Ots04q**	**Og04b**	**Ok14b**	**On27a**
2.2	*Cclu04a?*	**Ssa11a**	BC28	Omy26	**Co08b**	**Ots12q**	**Og05b**	**Ok02b**	**On28a**
3.1	Cclu25	Ssa14a	BC22	Omy08b	Co30	Ots10q	Og22	Ok23	On29
3.2	Cclu26	Ssa03a	BC11	Omy28	Co27	Ots28	Og08a	Ok15	On20b
4.1	Cclu16	Ssa09b	BC33	Omy25a	Co15a	Ots08q	Og23a	Ok30a	On05a
4.2	Cclu29	Ssa05a	BC07b	Omy14b	Co19b	Ots21	Og13a	Ok04b	On06b
5.1	Cclu05a	Ssa19b	BC01a	Omy16a	Co20a	Ots24	Og11b	Ok03	On23b
5.2	Cclu15	Ssa28	BC27	Omy20	Co25	Ots25	Og07a	Ok24	On16
6.1	*Cclu05b?*	Ssa01b	BC01b	Omy23	**Co11a**	**Ots01q**	**Og06b**	**Ok02a**	**On28b**
6.2	*Cclu05b?*	Ssa18a	BC36	Omy01b	**Co04b**	**Ots06q**	**Og08b**	**Ok36**	**On10a**
7.1	Cclu13	Ssa13b	BC08b	Omy12a	Co06a	Ots09p	Og16b	Ok12	On22b
7.2	Cclu08	Ssa04b	BC09	Omy10a	Co28	Ots30	Og02b	Ok11	On10b
8.1	Cclu36	Ssa23	BC04a	Omy04a	Co10a	Ots01p	Og06a	Ok26	On17
8.2	Cclu06a	Ssa10a	BC17	Omy05b	Co13a	Ots05q	Og15a	Ok01b	On24a
9.1	Cclu06b	**Ssa02b**	BC42	Omy13a	**Co20b**	**Ots32**	**Og26a**	**Ok32a**	**On21a**
9.2	Cclu38b	**Ssa12a**	BC03b	Omy17b	**Co01b**	**Ots02q**	**Og18a**	**Ok10b**	**On15b**
10.1	Cclu10	Ssa27	BC23	Omy18b	Co17a	Ots13p	Og24a	Ok20	On04a
10.2	Cclu24a	Ssa14b	BC34	Omy14a	Co14b	Ots31	Og09b	Ok19a	On07a
11.1	Cclu18	**Ssa06a**	BC14	Omy13b	**Co10b**	**Ots27**	Og26b	**Ok32b**	**On21b**
11.2	*missing*	**Ssa03b**	BC08a	Omy12b^a^	**Co06b**	**Ots09q**	Og16a	**Ok05**	**On26**
12.1	Cclu27	Ssa13a	BC18	Omy16b	Co24	Ots22	Og10a	Ok27	On20a
12.2	Cclu14	Ssa15b	BC30	Omy09b	Co17b	Ots16q	Og03a	Ok28a	On07b
13.1	Cclu34	Ssa24	BC06b	Omy06a	Co03a	Ots04p	Og04a	Ok14a	On27b
13.2	Cclu37	Ssa20a	BC40	Omy11a	Co08a	Ots12p	Og05a	Ok25	On13b
14.1	Cclu04b	Ssa01c	BC13	Omy05a	Co23	Ots20	Og11a	Ok09	On04b
14.2	Cclu33	Ssa11b	BC10	Omy29^a^	Co29	Ots33	Og20	Ok06	On19
15.1	Cclu31	Ssa09a	BC35	Omy25b	Co14a	Ots08p	Og10b	Ok35a	On12a
15.2	Cclu22	Ssa01a	BC12	Omy19b	Co07b	Ots11q	Og17a	Ok29a	On18a
16.1	Cclu02 + 03	Ssa21	BC26	Omy22	Co26	Ots26	Og01b	Ok07	On01
16.2	Cclu32	Ssa25	BC24	Omy03b	Co02b	Ots03q	Og09a	Ok34a	On03a
17.1	Cclu38a	Ssa12b	BC03a	Omy17a	Co01a	Ots02p	Og18b	Ok10a	On15a
17.2	Cclu21	Ssa22	BC21	Omy07b	Co05b	Ots07q	Og03b	Ok21	On02b
18.1	Cclu40	Ssa15a	BC19	Omy08a	Co12a	Ots05p	Og12a	Ok19b	On24b
18.2	Cclu17	Ssa06b	BC31	Omy04b	Co21	Ots18	Og14a	Ok08	On13a
19.1	Cclu30	Ssa10b	BC15	Omy02b	Co22	Ots19	Og01a	Ok17	On09
19.2	Cclu11	Ssa16a	BC20	Omy01a	Co04a	Ots06p	Og02a	Ok22	On25
20.1	*Cclu01a*^a^*?*	**Ssa05b**	BC07a	Omy02a	**Co13b**	**Ots23**	Og15b	**Ok31**	**On14a**
20.2	*Cclu01a*^a^*?*	**Ssa02a**	BC29	Omy03a	**Co02a**	**Ots03p**	Og19a	**Ok34b**	**On03b**
21.1	Cclu12	Ssa29	BC05b	Omy15a	Co11b	Ots29	Og07b	Ok04a	On06a
21.2	Cclu39	Ssa19a	BC16	Omy11b	Co18b	Ots16p	Og12b	Ok28b	On22a
22.1	*Cclu19?*	**Ssa17a**	BC39	Omy07a	**Co05a**	**Ots07p**	Og21b	**Ok37**	**On02a**
22.2	*Cclu19?*	**Ssa16b**	BC05a	Omy18a	**Co16b**	**Ots14p**	Og23b	**Ok30b**	**On05b**
23.1	Cclu02^a^	**Ssa07b**	BC02b	Omy21a	**Co09a**	**Ots15p**	Og25a	**Ok13b**	**On08b**
23.2	Cclu01b^a^	**Ssa17b**	BC37	Omy15b	**Co19a**	**Ots17**	Og14b	**Ok33**	**On23a**
24.1	Cclu24b	Ssa07a	BC02a	Omy21b	Co09b	Ots15q	Og25b	Ok13a	On08a
24.2	Cclu23	Ssa18b	BC32	Omy09a	Co16a	Ots10p	Og21a	Ok35b	On12b
25.1	*missing*	**Ssa04a**	BC04b	Omy10b	**Co12b**	**Ots34**	Og24b	**Ok16**	**On11b**
25.2	*missing*	**Ssa08**	BC41	Omy19a	**Co07a**	**Ots11p**	Og17b	**Ok29b**^a^	**On18b**

Note.—All orthologous relationships among salmonids and the pre-duplicated Northern Pike are displayed as identified by MapComp. Bold/gray-shaded species are those with recent studies providing evidence for residual tetraploidy using duplicate markers or sequence similarity, and within these species, the bold/gray-shaded homeologs are those with evidence of residual tetraploidy in the original studies (see table 1 for Coho, Chinook, Chum and Sockeye Salmon; Atlantic Salmon residual tetraploidy in the main regions identified consistently in Lien et al. 2011, 2016). Note that Northern Pike only has 25 chromosomes, but here each chromosome is listed twice to accommodate the duplicate orthologs in the other species. Note that Omy29 is referred to as OmySex in the original publication.

a Evidence for orthology is weak; italics in Lake Whitefish indicate problems resolving orthology.

In Brook Charr, a total of 8 metacentric and 34 acrocentric chromosomes were expected from salmonid cytogenetics (Lee & Wright 1981; Phillips & Ráb 2001) and all were identified here (table 2), increasing the resolution of the Brook Charr linkage maps from the existing low-density linkage maps constructed with microsatellites (*n* = 133 markers; Timusk et al. 2011) and expressed sequence tag SNPs (*n* = 266 markers; Sauvage et al. 2012a). Since Brook Charr has the fewest metacentric chromosomes of the species characterized here, often two acrocentric chromosomes in Brook Charr correspond to two fused arms on the same metacentric chromosome in another species. In some cases, due to multiple, tandem chromosome fusions observed in Atlantic Salmon, where one chromosome contains three chromosome arms (Phillips et al. 2009), three linkage groups in Brook Charr correspond to one linkage group in Atlantic Salmon. For example, Brook Charr BC33, BC35, BC38 are in tandem fusions in Atlantic Salmon Ssa09 (supplementary file S5, Supplementary Material online). We compared homologous chromosome arm relationships identified with MapComp (identical and proximate markers) between Coho Salmon and Chinook Salmon with results that used only homologous markers (Kodama et al. 2014) and found the same correspondence between these species. All Rainbow Trout and Coho Salmon results were concordant between the MapComp analysis and Kodama et al. (2014) except that Kodama et al. report that Co08a corresponds to both Omy11p and q arms and that Co18b corresponds to Omy11p, whereas we find that Co08a corresponds to the first half of Omy11 (a), and Co18b to the second half of Omy11 (b) (table 2). For Atlantic Salmon, there were more discrepancies: MapComp identifies Co01a, Co07a, Co12b, Co20b, and Co25 as corresponding to Ssa12b, Ssa08, Ssa04a, Ssa02b, and Ssa28, respectively; Kodama et al. identify these chromosome arms as corresponding to Ssa02q, Ssa04p, Ssa08q, Ssa12qa, and (Ssa08p + Ssa28), respectively. In some cases, there are discordances with the p and q designation with the first and second arm present in the linkage map (a and b designation). The reasons for these discrepancies are not clear, but it is worth noting that the two studies used different Atlantic Salmon genetic maps for comparison. This highlights the importance of meta-analyses to collect and analyze this data; here we refer to most arms as the Northern Pike homologs and include the Oxford grids to view the correspondence of all species against Brook Charr in supplementary file S5, Supplementary Material online, permitting the inference of these relationships directly from the data. The rest of the results among these species corresponded between the studies (total = 50 homologous chromosome arm relationships in four species). Additionally, chromosome arm homology was determined for Pink Salmon, Chum Salmon, Sockeye Salmon and Lake Whitefish.

### Homeologous Chromosome Arm Identification

To identify homeologous chromosome arms (i.e. chromosome arms originating from the same pre-duplicated chromosome), the genetic map of Northern Pike was compared with the maps of all species using MapComp as described in the Methods. All homeologous pairs in Atlantic Salmon identified by MapComp using the Rainbow Trout intermediate reference genome were concordant with those originally identified (Rondeau et al. 2014), but here were also extended to all other species (table 2).

Homeologous chromosome arms exhibiting residual tetraploidy can be identified by mapping duplicate markers in haploid crosses (e.g. Atlantic Salmon, Coho Salmon, Chinook Salmon, Sockeye Salmon and Chum Salmon; see table 1) or by sequence similarity (Lien et al. 2016). It has been observed that the same eight homeologous chromosome arm pairs exhibit residual tetraploidy in Coho Salmon, Chinook Salmon, Sockeye Salmon and Rainbow Trout (Brieuc et al. 2014; Kodama et al. 2014; Larson et al. 2016). Evidence for residual tetraploidy for some of these homeologous chromosome arms was identified from mapping studies (Lien et al. 2011), but more recently using sequence similarity in the genome assembly (Lien et al. 2016). Chum Salmon also exhibit residual tetraploidy in 16 chromosome arms although these were not yet integrated with the other species (Waples et al. 2016). As indicated by the homologous relationships in table 2 these are the same homeologous chromosome arm pairs. By inspecting the sequence similarity calculated between the homeologous chromosome arms in the Atlantic Salmon genome (Lien et al. 2016), and using the homologous relationships in table 2, it is apparent that one of the homeologous chromosome arm pairs expected to exhibit residual tetraploidy from the *Oncorhynchus* species is not in fact residually tetraploid in Atlantic Salmon (i.e. 18qa-1qa or Ssa18a-Ssa01b).

Without a haploid cross for our Brook Charr map, here we cannot specify whether any homeologs exhibit residual tetraploidy in this species. However, using MapComp, all chromosome arm homeologous relationships (total = 25 pairs) in all evaluated species were also identified for each species against Northern Pike using MapComp, with the exception of the aforementioned unidentifiable chromosome arms of Lake Whitefish (table 2). Interestingly, all of the missing homologous chromosome arm relationships in Lake Whitefish are, without exception, those exhibiting residual tetraploidy in the *Oncorhynchus* species (table 2). In several cases, the two homeologous chromosome arms from other species correspond to a single linkage group in Lake Whitefish (e.g. Sockeye Salmon 10a and 28b correspond to Lake Whitefish 05b). This observation may indicate pseudolinkage in these few cases in the Lake Whitefish genetic map, or could originate from another unknown issue. Pseudolinkage is statistical linkage between markers that should map to two separate linkage groups, and is a specialized case of residual tetrasomy where pairing of telomeric homologous regions preferentially occurs with the homeolog from the same genetic background in individuals with mixed genetic background (Ostberg et al. 2013; May & Delany 2015). This is a possible explanation for lack of differentiation of these chromosome arms in the Lake Whitefish genetic map, as the two populations of the cross were from two different post-glacial lineages (Gagnaire et al. 2013). Further study would be required to confirm this, but regardless, these chromosome arms remain difficult to identify in the Lake Whitefish genetic map.

### Conserved and Species-Specific Chromosome Rearrangements

**Fig. 4.—:**
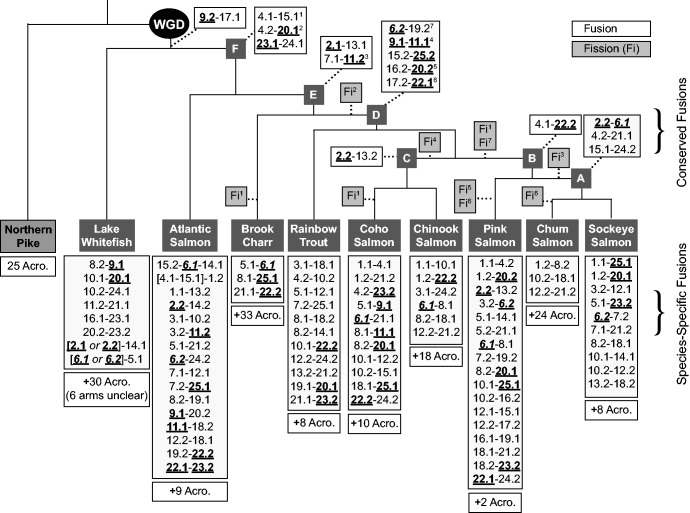
Fusions and fissions across the salmonid lineage. Different fusions and fissions have occurred during the evolution of the salmonids. White boxes display the fusion events, where the homologous chromosomes for all species are named according to the corresponding Northern Pike linkage group ID, with.1 or.2 to correspond with the post-duplicated salmonid chromosomes. Bold and underlined chromosome numbers are the homeologous pairs that exhibit residual tetraploidy, and the italicized chromosome (6.1/6.2) does not exhibit residual tetraploidy in Atlantic Salmon but does in the Pacific salmonids. Above the species names are conserved fusions, whereas below are the species-specific fusions. Also shown are the most likely timings of fissions in light gray boxes with the notation Fi^x^, where *x* corresponds to the superscript number on the original fusion. For example, the fusion 4.2–20.1^2^ at point (F) in the phylogeny probably underwent fission at Fi^2^ prior to D. The phylogeny is adapted from (Stearley & Smith 1993; Kinnison & Hendry 2004; Crête-Lafrenière et al. 2012), with minor modifications to the relationships within the Pink, Chum and Sockeye Salmon clade, as described in the results. Branch lengths are not to scale and are for illustrative purposes of relationships between species only.

Shared rearrangements among species in a clade (e.g. fusion events) are likely to have occurred prior to the diversification of the clade, as demonstrated for nine metacentric fusions in Coho Salmon and Chinook Salmon (Kodama et al. 2014). Here, chromosome arm correspondence furthered this analysis, allowing the inclusion of Brook Charr and Lake Whitefish as well as the clade containing Sockeye Salmon, Pink Salmon and Chum Salmon within *Oncorhynchus.* We identified 16 different fusion events conserved in at least 2 species, 5 fission events conserved in at least 2 species, 87 species-specific fusion events, and 5 species-specific fission events (fig. 4). For clarity, when discussing fusions and fissions here we use chromosome names from the Northern Pike chromosomes to refer to chromosome arms, including the duplicate designation (e.g. 1.1/1.2 or 3.1/3.2), as shown in table 2. The phylogeny in figure 4 was adapted from previous literature (Stearley & Smith 1993; Kinnison & Hendry 2004; Crête-Lafrenière et al. 2012). For the clade containing Pink, Sockeye and Chum Salmon, in which the sister relationships remain unclear (Kinnison & Hendry 2004), we present the most parsimonious phylogenetic relationship in terms of required number of fusion/fission events. With Pink Salmon as the sister species to the Chum and Sockeye Salmon clade, this requires three fewer fission or fusion events, and one fewer fission of a conserved metacentric chromosome, the 7.1–11.2 fusion.

The oldest identified rearrangement is the 9.2–17.1 fusion event that is conserved in all species investigated (see fig. 4). Conserved in all species except the basally diverging Lake Whitefish is the 23.1–24.1 fusion (see F in fig. 4). Another metacentric fusion event at this same point in the phylogeny was also identified (4.2–20.1) that is still present in both Atlantic Salmon and Brook Charr, but not in any members of *Oncorhynchus* (the species after D in fig. 4), suggesting that a fission occurred prior to the speciation of any members of *Oncorhynchus* (i.e. at Fi^2^ in fig. 4). One fusion (2.1–13.1) is present in Brook Charr and all *Oncorhynchus* spp. (fused at E in fig. 4). Another fusion at this same point (7.1–11.2; E in fig. 4) is found in all descendants except for the Chum and Sockeye Salmon clade (fission Fi^3^ in fig. 4).

More recent rearrangements include five fusions prior to the speciation of the *Oncorhynchus* clade (D in fig. 4). One is present in all *Oncorhynchus* species (15.2–25.2), another is present in all *Oncorhynchus* species except Pink Salmon (16.2–20.2; see Fi^5^ in fig. 4), another underwent fission in the Chinook/Coho lineage (9.1–11.1; see Fi^4^) and one underwent fission in Pink and Chum Salmon (17.2–22.1; see Fi^6^). Conserved fusions were found within the *Oncorhynchus* lineage as well, including one fusion in the Chinook/Coho lineage, (2.2–13.2; C in fig. 4) and three fusions prior in the Sockeye/Chum lineage (A in fig. 4). Each species also has had species-specific fusions, ranging in number from only three fusions in Chum Salmon and three in Brook Charr to up to 17 in Pink Salmon and 18 in Atlantic Salmon (fig. 4).

Some rearrangements are more complex and thus it is more difficult to unambiguously describe their history. For example, a triple chromosome arm fusion in Atlantic Salmon occurred by an initial metacentric fusion (4.1–15.1) followed by an Atlantic Salmon-specific fusion of this metacentric chromosome [4.1–15.1] with an additional acrocentric chromosome, 1.2. The initial 4.1–15.1 fusion either fused once prior to the divergence of Atlantic Salmon and underwent three different fission events (Fi^1^ in fig. 4), or fused three independent times with the same fusion partner. It is not clear which of these possibilities is correct, but in figure 4, we display the first and more parsimonious scenario. In Atlantic Salmon, after the metacentric fusion, an additional fusion occurred, adding a third chromosome arm (1.2 with [15.1–4.1]). Three other different fusions appeared to have occurred two independent times: 8.2–18.1 in Chinook and Sockeye; 12.2–21.2 in Chinook and Chum; and 7.2–25.1 in Atlantic Salmon and Rainbow Trout. For each of these multiple independent origins, the alternate explanations are possible but less parsimonious. Although these few independent origin cases are not entirely clear, we display the most parsimonious rearrangements, requiring the fewest independent fusions/fissions in figure 4.

### Putative Lineage-Specific Inversions

**Fig. 5.—:**
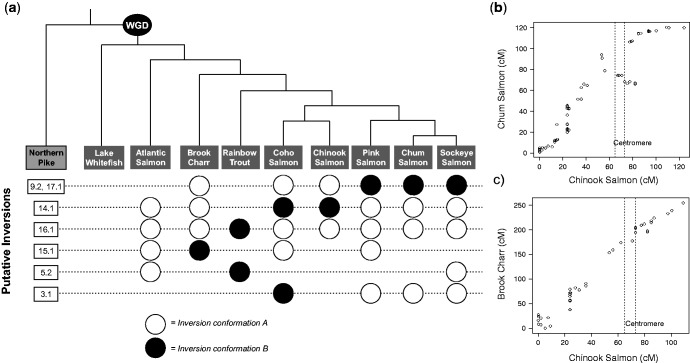
Putative conserved and species-specific inversions. (*a*) The salmonid phylogeny is shown highlighting six different inversion events, each listed according to the Northern Pike chromosome and represented on a dotted line below the phylogeny. Per line (inversion event), white circles indicate the more common or likely ancestral inversion conformation, and black circles the less common and more likely derived inversion conformation. If no circle is present, the inversion was not visible in the linkage map comparison of that species. The putative pericentric inversion across the fusion 9.2–17.1 is displayed in (*b*) showing two species (Chum and Chinook Salmon) with different inversion conformations, and in (*c*) for two species with the same conformation (Brook Charr and Chinook Salmon). Predicted centromere positions previously identified in Chinook Salmon (Brieuc et al. 2014) are also shown in (*b* and *c*). Full names for species are defined in table 1, the phylogeny is as described in the “Results” section and figure 4, and probable genes within the 9.2–17.1 inversion are shown in supplementary file S4, Supplementary Material online.

Several inversions flanked by noninverted regions were revealed between linkage maps, suggesting the presence of chromosomal segment inversions (fig. 5). These putative inversions are more supported when phylogenetically conserved. Future genome assemblies for the species involved will be valuable for further inversion identification. A striking putative inversion was identified in one of the metacentric chromosomes conserved across all evaluated salmonids (9.2–17.1; fig. 5). An inversion near the center of the linkage group is present in only the Pink, Chum and Sockeye Salmon lineage. This inversion is visible in the Oxford grids between these species and Coho, Chinook Salmon and Brook Charr (see fig. 5*a* and *b*). As a result, the conformation observed in Pink, Chum and Sockeye Salmon is likely the derived form. Rainbow Trout does not indicate the inversion against the ancestral conformation, but also does not indicate the inversion with Pink, Chum and Sockeye Salmon, as there is a gap with no marker pairs available at the inverted locus in the Rainbow Trout linkage group.

To further characterize the 9.2–17.1 inversion, centromere locations obtained from Chinook Salmon (Brieuc et al. 2014) were compared with the location of the inversion. The inverted region corresponds to the Chinook Salmon linkage group Ots02 between ∼49–82 cM (see fig. 5*b*) and the centromere for this linkage group was estimated to be between 65 and 73 cM. This inversion therefore most likely contains the centromere (i.e. a pericentric inversion). This inverted region between Chinook Salmon and Chum Salmon was visible when using either the Rainbow Trout or Atlantic Salmon genome as the intermediate reference genome, and had evidence from 12 markers mapping through two different scaffolds. To identify the genes that may be contained within this inverted region, the mapped locations on the Atlantic Salmon genome of the markers at the distal ends of the inverted region were taken. As the Atlantic Salmon genome has been annotated (Lien et al. 2016), this region of the genome (Ssa12 between 37,048,324 and 44,754,074 bp) was inspected for gene content. This region (∼7.7 Mb) putatively contains 11 genes (based on alignment evidence, here we do not include predicted genes), including *cytokine-like protein 1*, *solute carrier family 2, facilitated glucose transporter member 9* and *cd8 beta*, among others (see supplementary file S4, Supplementary Material online). Genes contained within an inversion are important because these areas have suppressed recombination (Ostberg et al. 2013) and the disruption of synteny can affect the regulatory environment of the genes. The exact genes found in this region in the species with the derived conformation of the inversion will require more genomes to be available before exploring further, including the actual breakpoints of the inversion and whether these occur within coding genes. In summary, 9.2 fused with 17.1 in the ancestor of all salmonids investigated here, then an inversion of a segment ∼7.7 Mb containing coding sequences occurred across the centromere specifically in the Pink, Chum and Sockeye Salmon lineage. Other inversions were also visible (fig. 5*a* and supplementary file S5, Supplementary Material online). More information on these and potentially new inversions will be obtained as more assembled genomes become available.

### Benefits of MapComp versus Direct Marker Comparison and Effect of Intermediate Reference Genome

Linkage group homology between species are typically identified by finding homologous markers using reciprocal best-hit blast (Kodama et al. 2014). The method implemented in MapComp, where we accept both identical and proximate (nearby) markers, leads to a far greater number of retained marker pairs (on an average 5-fold; table 3). For example, between Brook Charr and Chinook Salmon, 907 marker pairs were identified using MapComp, whereas direct mapping identified 190 pairs. Based on the clear correspondence along linkage groups, the pairs connected by MapComp add a significant number of markers without substantial random pairing (i.e. randomly placed points on the Oxford grids).

**Table 3:** MapComp Results Using Two Different Intermediate Genomes and Comparison with Results from Reciprocal blast

Species	Total Markers	Map to Rainbow Trout (RT) Genome: No. (%)	No. Marker Pairs (RT)	Map to Atlantic Salmon (AS) Genome: No. (%)	No. Marker Pairs (AS)	Recip. Best-Hit blast
Lake Whitefish	3,438	1,156 (33%)	346	1,185 (34%)	609	111
Atlantic Salmon	5,650^a^	2,776 (49%)	619	3,434 (60%)	1,041	208
Brook Charr	3,826	2,321 (60%)	N/A	2,454 (64%)	N/A	N/A
Rainbow Trout	955	837 (87%)	300	626 (65%)	411	30
Coho Salmon	5,377	3,873 (72%)	813	2,856 (53%)	1,068	182
Chinook Salmon	6,352	4,663 (73%)	907	3,472 (54%)	1,162	190
Pink Salmon	7,035	4,544 (65%)	841	3,481 (49%)	1,129	210
Chum Salmon	6,119	4,150 (67%)	795	3,139 (51%)	1,049	205
Sockeye Salmon	6,262	4,034 (64%)	771	3,138 (50%)	1,061	209

Note.—The number and percentage of markers from each species that map to each genome are shown, along with the number of markers pairs between each species and Brook Charr identified by MapComp. Also shown is the number of homologous markers that would have been found between each species and Brook Charr with a reciprocal best-hit blast approach. Numbers of markers mapping and being paired were similar when tested on the Rainbow Trout (RT) or Atlantic Salmon (AS) genome assemblies. *N/A* values are present as Brook Charr is not paired against itself.

a From EST sequences.

MapComp was tested on both the Rainbow Trout and the Atlantic Salmon genome as intermediate references for pairing markers between maps (table 3). Results using either genome were highly concordant and indicated all of the same chromosome homology (see supplementary files S5, Supplementary Material online), except in one instance where using the Rainbow Trout genome incorrectly identified that Brook Charr LG29 corresponds to Ssa05b, whereas in fact it corresponds to the other homeolog, Ssa02a. The reason for this one discrepancy between the two reference genomes is not clear. However, other than this there were only minor differences in the number of markers mapped and paired; although the Rainbow Trout genome as an intermediate provided slightly more mapped markers (on an average 1.2-fold more than Atlantic Salmon), the Atlantic Salmon genome provided more marker pairs (on an average 1.4-fold). Higher numbers of markers mapping to the Rainbow Trout is probably due to the closer phylogenetic distance to the majority of the species compared, and the higher number of marker pairs is probably due to the increased contiguity of the Atlantic Salmon genome. It is worth noting regarding these comparisons that the Atlantic Salmon linkage map sequence information was obtained in EST format, and therefore the number of markers mapping from the map to the genome was lower than expected relative to the shorter reads, probably due to the longer sequences and because *BWA mem* is not a splice-aware aligner.

MapComp parameters can be adjusted for the maximum distance allowed between paired markers. Here we used a maximum distance of 10 Mbp, but most paired markers were at a much smaller distance than this (see marker distance distribution examples in supplementary file S6, Supplementary Material online). With a greater phylogenetic distance between species, fewer identical markers were expected (Gonen et al. 2015). The number of identical markers for Chinook and Coho Salmon are high, but the Chinook Salmon comparison with Brook Charr depended more on nonidentical markers, as did the comparison between Lake Whitefish and Brook Charr. The distance parameter can be easily tested by the user, allowing the identification of the optimal settings for individual datasets to permit the greatest number of paired marker comparisons without increasing off-target pairing in the Oxford grids. Here we found that 10 Mbp provided more markers without a substantial increase in noise.

## Discussion

Linkage maps have many applications, including QTL analysis, assisting genome assembly and comparative genomics. With advances in sequencing technology and techniques (Baird et al. 2008), high quality and dense linkage maps are increasingly available for many species, including nonmodel species. Dense linkage maps are highly useful for anchoring genome scaffolds to chromosomes (Ming & Man Wai 2015) or for comparative genomics, allowing for information transfer from model to related nonmodel organisms (Naish et al. 2013). They are also useful for cross-species QTL comparisons (Larson et al. 2016) to understand genome function and evolution, such as that after a whole genome duplication (Kodama et al. 2014).

Salmonids are a valuable taxon for studying genome duplication. Recently, Kodama et al. (2014) characterized several chromosomal fusions and positioned them in the salmonid phylogeny based on the conservation of the fusion across the investigated lineages. This has indicated that structural rearrangements have occurred throughout the evolution of the salmonids, with rearrangements retained from different points in evolutionary history. Here, we further demonstrate the diversity of these rearrangements by identifying all homologous arms in additional species and genera, and evaluate the most likely timings of rearrangements throughout salmonid evolutionary history.

At least one homeologous chromosome arm fused into a metacentric chromosome is thought to be required for recombination between homeologs (Kodama et al. 2014). This is important to consider for salmonid genome assembly. If a homeologous chromosome pair that is known to exhibit residual tetraploidy in other salmonid species (see table 2) occurs as two acrocentric chromosomes in another species, this pair may not be residually tetraploid and therefore may have increased sequence divergence between homeologs. Therefore, species with few, or none of the chromosomes in metacentric fusions may offer additional information regarding the salmonid rediploidization process. Even though Brook Charr has fewer metacentrics than other salmonids, all of the known residually tetraploid homeolog pairs from the other salmonids have one homeolog present in a metacentric chromosome in Brook Charr (table 2; *n* = 8 metacentrics).

As metacentric formation is thought to be important for ongoing recombination between homeologs, the timing of fusion events may provide additional insight into the rediploidization process in salmonids. From the present study, it is interesting to note that many of the residually tetraploid pairs have at least one homeolog involved in an ancient conserved fusion (fig. 4). The second homeolog varies more in its fusion partner across the lineage, or can be present as an acrocentric. For example, 9.2 of the 9.1/9.2 residually tetraploid homeolog pair is fused with 17.1 in all assessed species (fused at F in fig. 4). In contrast, 9.1 varies more in its binding partner and sometimes is acrocentric in extant salmonids. Similarly, the residually tetraploid 23.1 is fused with 24.1 in all assessed species except Lake Whitefish (10.2–24.1), whereas 23.2 is more variable and occasionally acrocentric. These ancient fusions may be informative about mechanisms that have prevented rediploidization in salmonids.

The fusion history of the other residually tetraploid pairs are not as simple as the above two examples. Within a residually tetraploid homeolog pair, it is not always the same homeolog in a metacentric fusion across species. This agrees with previous indications that only one of the homeologs must be bound in a metacentric to prevent rediploidization. For example, in Atlantic Salmon, 2.2 is metacentric and 2.1 is acrocentric, whereas in Brook Charr and all *Oncorhynchus* spp., 2.1 is the conserved metacentric (fused at E in fig. 4). Another example of this differing metacentric binding occurs for 20.1/20.2, where in Atlantic Salmon and Brook Charr 20.1 is in a conserved metacentric fusion and 20.2 is acrocentric, whereas in all *Oncorhynchus* spp. 20.2–16.2 is the conserved fusion. Therefore, even though these metacentrics may be required to retain residual tetraploidy, the homeolog bound in the metacentric chromosome can differ among the species. Further characterization of this will be facilitated with the production of high-density linkage maps for more species from salmonid genera outside of *Oncorhynchus* that are represented in the present work by only one species (e.g. *Salvelinus*, *Coregonus*, *Salmo*) or none (e.g. *Thymallus*).

Although rediploidization of the salmonids may have generally occurred prior to the salmonid radiation (Lien et al. 2016), the rate of rediploidization has varied across the lineages since the speciation of Atlantic Salmon at least in one homeologous pair (i.e. 6.1/6.2). This pair has rediploidized in *Salmo salar* as demonstrated by sequence similarity between homeologous chromosomes (see 18qa-1qa in Figure 3b in Lien et al. 2016) as well as suggested by the lack of identifiable isoloci in this pair (Lien et al. 2011). Conversely, in *Oncorhynchus*, this homeologous pair exhibits residual tetraploidy as demonstrated by isoloci in Coho, Chinook, Chum and Sockeye Salmon (see table 1 for references). Identification of this difference in rediploidization was possible due to the comprehensive characterization of the homologous relationships of chromosome arms (table 2) and the previous studies documenting residual tetraploidy (Brieuc et al. 2014; Kodama et al. 2014; Larson et al. 2016; Lien et al. 2016; Waples et al. 2016). It is interesting to note that one of these homeologous chromosome arms that does not exhibit residual tetraploidy in Atlantic Salmon (i.e. 6.1) is fused in the center of one of the triple chromosome arm fusions specific to Atlantic Salmon (fig. 4). Although it is known that residual tetraploidy requires at least one of the two homeologous chromosomes to be in a metacentric fusion, the effect of being in the middle of a triple chromosome arm fusion on rediploidization is not known. It is possible that this position could hinder homeologous pairing at meiosis. Regardless of the mechanism, this result indicates that the path to rediploidization differs for this chromosome pair between Atlantic Salmon and the evaluated Pacific salmonids. Information regarding residual tetraploidy (e.g. through haploid crosses) in additional maps from members of *Coregonus*, *Salvelinus*, *Thymallus* or other salmonid genera will be valuable to understand this process further in a broader range of genera.

### Fusions and Inversions

Chromosomal rearrangements include chromosome fusions or fissions, region amplifications or deletions, segment inversions or nonhomologous chromosome segment translocations (Rieseberg 2001). The characterization of the fusion events across all published salmonid maps (fig. 4) provides a new resolution of the exact identities of chromosome arms in the pre-duplicated genome that have fused together at different moments during the salmonid diversification. This demonstrates the stepwise process of generating the extant salmonid karyotypes, with fusions occurring at each step along the diversification process. Notably, for most salmonid species, most fusions are not ancestrally conserved, but rather occur individually within each species (fig. 4). It remains unclear why some species retain their high number of acrocentric chromosomes (e.g. Brook Charr, 3 species-specific fusions), whereas others do not (e.g. Pink Salmon, 17 species-specific fusions). Furthermore, this variation in numbers of species-specific fusions can occur between closely related species (e.g. Chum and Sockeye Salmon).

Inversions can occur when a segment of a chromosome is cut out by two breakpoints and then reinserted in the opposite orientation (Kirkpatrick 2010). Effects of inversions on fitness are highly unpredictable and vary across taxa. In general, they tend to reduce recombination rates at the site of the inversion, potentially playing an important role in speciation and local adaptation (Noor, et al. 2001a; 2001b; Rieseberg 2001; Kirkpatrick 2010). For example, two inversions reduce recombination and maintain genetic differentiation between migratory and stationary ecotypes of Atlantic Cod (*Gadus morhua*), preserving the co-occurrence of adaptive alleles within the migratory form (Kirubakaran et al. 2016) Additionally, lower recombination rates were observed in heterokaryotypic regions of Yellowstone Cutthroat Trout (*O. clarkii*) and Rainbow Trout hybrids compared with collinear regions (Ostberg et al. 2013). Recombination suppression may allow for conservation of fitness-related gene complexes that are locally adapted, or involved in reproductive isolation (Ostberg et al. 2013). Robertsonian rearrangements (e.g. fusions and fissions) have less of an effect on recombination rates than do rearrangements affecting synteny (e.g. inversions) (Rieseberg 2001; Ostberg et al. 2013). It is possible that an inversion occurring within a region of residual tetraploidy could result in a reduction of recombination between the homeologs; the relationship between inversions and residual tetraploidy merits further exploration.

### MapComp: Potential and Limitations

By using the information from both identical and proximate marker pairing, MapComp helps to solve the issue of low marker homology between reduced representation sequencing (e.g. RADseq) based linkage maps generated with different protocols or restriction enzymes, or from relatively more distantly related species. Synteny is still required in order to pair proximate markers through the intermediate reference genome. Previously, polymorphic microsatellite markers highly conserved among salmonids have enabled exploration of salmonid chromosomal evolution by integrating across species and genera (Naish et al. 2013). Although RADseq-based linkage maps routinely provide an order of magnitude more markers than microsatellite maps with less effort, identical markers are not always abundant between species. Low marker homology among species has also hindered cross-species comparisons when using microsatellite-based genetic maps, for example when Coho Salmon was compared with Sockeye Salmon and Pink Salmon (Naish et al. 2013). As such, with the generation of additional high-density maps for the salmonids, the use of MapComp will continue to be highly useful in characterizing these relationships.

At its core, MapComp is similar to the approach used by Sarropoulou et al. (2008), in which EST-based markers from two species were aligned to a reference genome of a third species, to identify homologous linkage groups. However, this earlier approach did not retain marker positions from original maps for plotting within an Oxford grid, and only provided the total number of markers found to correspond for each linkage group pair. Other cross-species map comparison approaches exist, for example cMAP (Fang et al. 2003), although these often require shared markers between maps. Another similar approach was used by Amores et al. (2011) for Spotted Gar *Lepisosteus oculatus*, where paired-end sequencing was performed on a single-digest then randomly sheared library. The authors therefore obtained a larger amount of sequence near their marker allowing them to identify genes near the marker. Then the order of the identified genes was used to compare synteny of homologs in assembled genomes such as humans *Homo sapiens* or Zebrafish *Danio rerio*. In contrast, MapComp works without prior knowledge of specific gene orthology, providing map comparisons at a much higher marker density without being restricted to coding regions. Another recent approach compared a linkage map for the European tree frog *Hyla arborea* with the genome of the western clawed frog *Xenopus tropicalis* and identified many syntenic regions (Brelsford et al. 2016). A recent approach in salmonids used a RADseq high-density linkage map for Chinook Salmon with the Atlantic Salmon reference genome to anchor Atlantic Salmon scaffolds to the Chinook Salmon linkage map when enough markers were present and the order was as expected (McKinney et al. 2016). Homologous relationships between Chinook Salmon and Atlantic Salmon have been characterized previously (Brieuc et al. 2014), and so the aligned scaffolds could then be classified as homologous, homeologous, or unsupported to further improve the anchoring of scaffolds onto the linkage map, and to identify potential genes around loci of interest (McKinney et al. 2016). MapComp is not meant to be used for RADseq based phylogenetic analysis, which requires identical markers for comparisons; this is rather performed using the direct marker approach with reciprocal best hit blast (Cariou et al. 2013; Pante et al. 2014).

MapComp is thus an easy solution to compare genetic maps in a way that is more tolerant of different library preparation protocols and phylogenetic distances. As shown here, MapComp is effective at finding homology between chromosomes (table 2), permitting the characterization of chromosomal rearrangements since whole genome duplication (fig. 4) and identifying putative structural rearrangements (fig. 5). This method will allow for the exploration of corresponding regions between species, such as regions harboring QTLs (Sarropoulou et al. 2008). Advances in genomics have resulted in many taxonomic groups having at least one species with a reference genome at some stage of assembly, providing the intermediate genome needed for this approach, and opening up this approach for a number of other taxonomic groups. MapComp is freely available at: https://github.com/enormandeau/mapcomp/

## Conclusions

We provide the most complete analysis to date of the chromosomal rearrangements that lead to the current chromosome conformations in salmonids using the newly developed MapComp method. This analysis permitted the integration of all high-density salmonid maps across the lineage, identifying the timing of fusions of all chromosomes, including those still undergoing residual tetraploidy in the characterized species. This comparative analysis confirmed the observation that the homeologous chromosome arm pairs exhibiting residual tetraploidy have at least one arm present in a metacentric fusion, although the specific homeolog may differ among lineages. Furthermore, we identified that a lineage-specific difference in rediploidization occurring specifically in Atlantic Salmon may be due to one of the two homeologous chromosome arms being in the center of a triple chromosome arm fusion and therefore possibly less accessible for recombination between the homeologs. Large inversions were also identified using MapComp, including a pericentric inversion that has occurred after the salmonid-wide conserved ancestral fusion of two chromosome arms that putatively rearranged the position of 11 genes across a centromere. These analyses will be further refined through the continued availability of other high-density salmonid maps, and can provide insights into the chromosomal evolution in both salmonids and other taxa.

## Code and Pipeline Availability

MapComp: https://github.com/enormandeau/mapcomp/

Collecting and formatting available salmonid maps: https://github.com/bensutherland/2016_ms_sfonmap

RADseq workflow: http://gbs-cloud-tutorial.readthedocs.org stacks workflow: https://github.com/enormandeau/stacks_workflow

## Supplementary Material

Supplementary files S1–S7 are available at *Genome Biology and Evolution* online (http://www.gbe.oxfordjournals.org/).

## Supplementary Material

Supplementary Data
