# Supplementary material for: Salmonid Chromosome Evolution as Revealed by a Novel Method for Comparing RADseq Linkage Maps
Source: Genome Biol Evol. 2016 Nov 9;8(12):3600–17. doi: 10.1093/gbe/evw262 (PMC5381510; doi:10.1093/gbe/evw262)
Supplement: Supplementary Data [file evw262_Supp.zip › additional_fileS6_distance_between_markers.pdf]

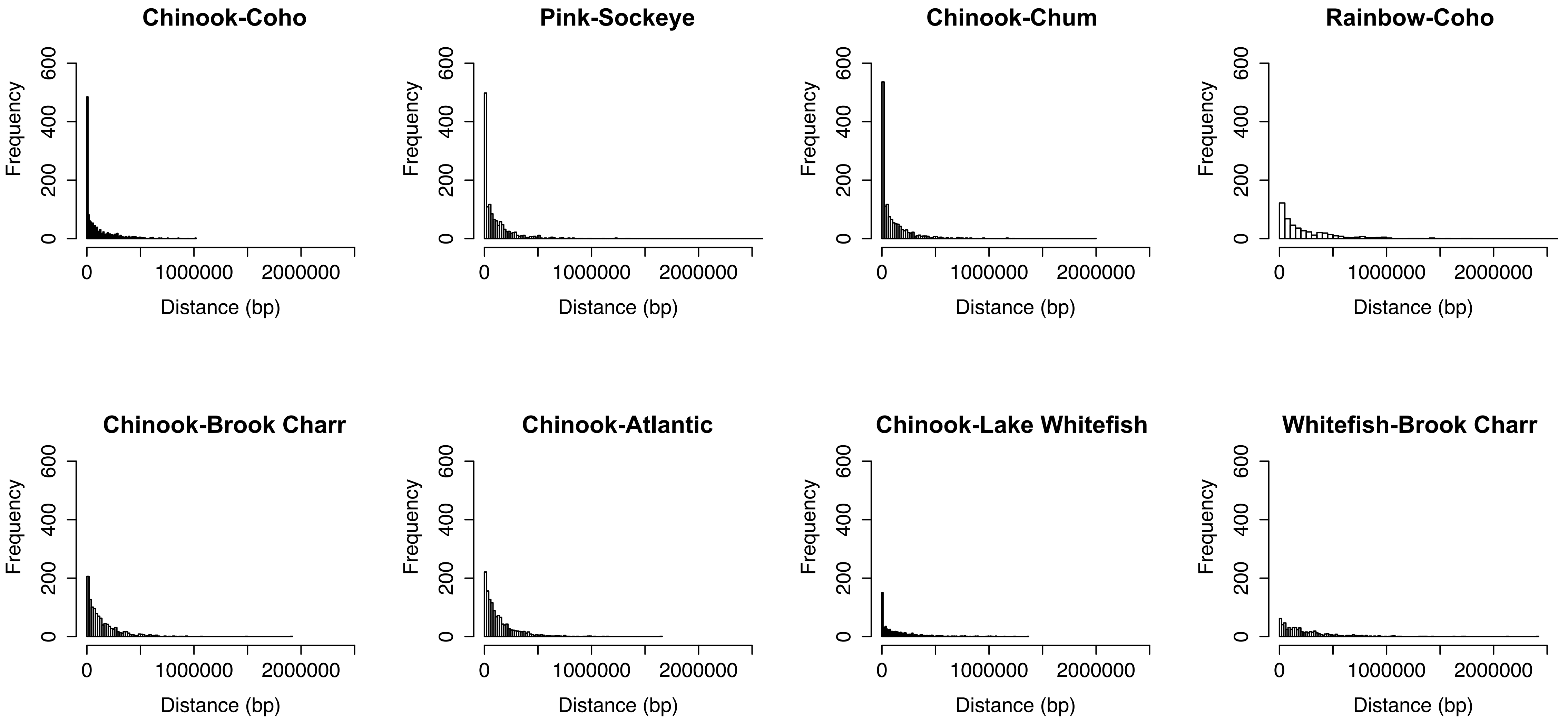
Chinook-Coho
Pink-Sockeye
Chinook-Chum
Rainbow-Coho
Chinook-Brook Charr
Chinook-Atlantic
Chinook-Lake Whitefish
Whitefish-Brook Charr
Frequency
0
200
400
600
0
1000000
2000000
Distance (bp)
