# Supplementary material for: Salmonid Chromosome Evolution as Revealed by a Novel Method for Comparing RADseq Linkage Maps
Source: Genome Biol Evol. 2016 Nov 9;8(12):3600–17. doi: 10.1093/gbe/evw262 (PMC5381510; doi:10.1093/gbe/evw262)
Supplement: Supplementary Data [file evw262_Supp.zip › additional_fileS1_sfon_building_parameters_2015-02-15.pdf]

Additional File 1.

STACKS parameters (Table 1), bioinformatics steps for Brook Charr map construction (Table 2) and JOINMAP parameters (Table 3).

Table 1. STACKS parameters.

Modules	Parameters	Value
process_radtags	Clean data by removing any read with an uncalled base (c)	Yes
	Discard reads with low quality scores (q)	Yes
	Truncate final read length to this value (t)	80
ustacks	Minimum depth of coverage required to create a stack (m)	4
	Max nucleotides distance allowed between stacks (M)	8
	Max distance allowed to align secondary reads to primary stacks (N)	10
	Disable calling haplotypes from secondary reads (H)	Yes
	Enable the removal algorithm, to drop highly-repetitive stacks (and nearby errors) from the algorithm (r)	Yes
	Enable the Deleveraging algorithm (d)	Yes
	Maximum locus stacks	3
	Bounded model with an alpha	0.05
	Lower and upper bound epsilon	0-0.05
cstacks	Number of mismatches allowed between sample tags when generating the catalog (n)	0
sstacks	Default	Default
rxstacks	Log liklihood filtering	Yes
	Minimum log likelihood threshold	-10
	Prune haplotypes	Yes
	Filter confounded loci	Yes
	Confounded threshold	0.75
	Bounded model with an alpha	0.1
	Lower and upper bound epsilon	0-0.05
genotypes	Minimum percentage of progeny to print a marker (r)	50-95%
	Make automated corrections to the data (c)	Yes

Table 2. Bioinformatic steps used to build the Brook Charr linkage map.

Step	Description	Software, version and module
**1**	De-multiplex and clean sequences	STACKS v.1.32 *process_radtags*
**2**	Sequences from each individual are grouped into stacks, and markers are identified	STACKS v.1.32 *ustacks*
**3**	Markers of the parents and offspring are grouped to form a parental catalogue	STACKS v.1.32 *cstacks*
**4**	Offspring markers are compared to parental catalogue to determine the allelic state of markers	STACKS v.1.32 *sstacks*
**5**	Genotype and haplotype calls in individual samples are corrected based on data accumulated from a population-wide examination (here family-wide).	STACKS v.1.32 *rxstacks*
**6**	Allelic states are translated into a *de novo* assembly to generate markers for positioning on the linkage map	STACKS v.1.32 *populations*
**7**	Generate a linkage map using a multipoint maximal likelihood algorithm	JOINMAP v.4.1

Table 3. JOINMAP parameters.

Parameters	Value
**Spatial sampling recombination frequency threshold:**	
1	0.1
2	0.05
3	0.03
4	0.02
5	0.01
Number of map optimization rounds per sample:	5
**Simulated annealing parameters (map order optimization):**	
Chain length (with constant acc.prob.)	2000
Initial acceptance probability	0.25
Cooling control parameter	0.001
Stop after # chains without improvement	10000
**Gibbs sampling parameters (MC-EM multipoint ML estimation of rec. frequencies):**	
Length of burn-in chain	10000
Number of EM cycles	10
Chain length per EM cycle	3000
Period between rec.freq. matrix samples	10
