# Supplementary material for: Salmonid Chromosome Evolution as Revealed by a Novel Method for Comparing RADseq Linkage Maps
Source: Genome Biol Evol. 2016 Nov 9;8(12):3600–17. doi: 10.1093/gbe/evw262 (PMC5381510; doi:10.1093/gbe/evw262)
Supplement: Supplementary Data [file evw262_Supp.zip › additional_fileS2_sfon_female_map_summary_stats.pdf]

Additional File 2

Table 1. Female Brook Charr linkage map summary statistics.

LG	Metacentric / Acrocentric	Distance (cM)	Number markers	Density (marker/cM)	*ab* x *ac*	*ab* x *ab*	*ab* x *aa*
Sf01	M	343	175	0.5	7	46	122
Sf02	M	318	107	0.3	13	18	76
Sf03	M	273	144	0.5	9	26	109
Sf04	M	267	110	0.4	12	24	74
Sf05	M	262	111	0.4	9	26	76
Sf06	M	258	136	0.5	14	26	96
Sf07	M	256	109	0.4	8	37	64
Sf08	M	185	112	0.6	11	33	68
Sf09	A	230	105	0.5	5	19	81
Sf10	A	224	124	0.6	15	24	85
Sf11	A	213	92	0.4	5	37	50
Sf12	A	199	101	0.5	6	27	68
Sf13	A	192	117	0.6	5	29	83
Sf14	A	172	52	0.3	10	3	39
Sf15	A	184	134	0.7	6	30	98
Sf16	A	181	90	0.5	5	12	73
Sf17	A	178	103	0.6	8	19	76
Sf18	A	177	103	0.6	8	27	68
Sf19	A	176	87	0.5	3	21	63
Sf20	A	175	114	0.7	3	32	79
Sf21	A	170	98	0.6	5	30	63
Sf22	A	166	102	0.6	5	38	59
Sf23	A	163	93	0.6	4	23	66
Sf24	A	162	95	0.6	4	15	76
Sf25	A	159	87	0.5	6	22	59
Sf26	A	157	70	0.4	4	20	46
Sf27	A	156	70	0.4	2	21	47
Sf28	A	150	78	0.5	5	16	57
Sf29	A	145	63	0.4	1	11	51
Sf30	A	144	85	0.6	4	29	52
Sf31	A	144	87	0.6	2	20	65
Sf32	A	143	79	0.6	5	22	52
Sf33	A	132	79	0.6	11	20	48
Sf34	A	132	83	0.6	2	25	56
Sf35	A	125	67	0.5	6	26	35
Sf36	A	123	53	0.4	1	19	33
Sf37	A	121	54	0.4	5	12	37
Sf38	A	119	74	0.6	8	21	45
Sf39	A	115	43	0.4	6	9	28
Sf40	A	101	73	0.7	2	28	43
Sf41	A	97	33	0.3	2	3	28
Sf42	A	65	34	0.5	2	8	24
Total	N/A	7452	3826	N/A	254	954	2618
Avg. per LG	N/A	177	91	0.5	6.0	22.7	62.3
