# Supplementary figures and images for: Salmonid Chromosome Evolution as Revealed by a Novel Method for Comparing RADseq Linkage Maps
Source: Genome Biol Evol. 2016 Nov 9;8(12):3600–17. doi: 10.1093/gbe/evw262 (PMC5381510; doi:10.1093/gbe/evw262)

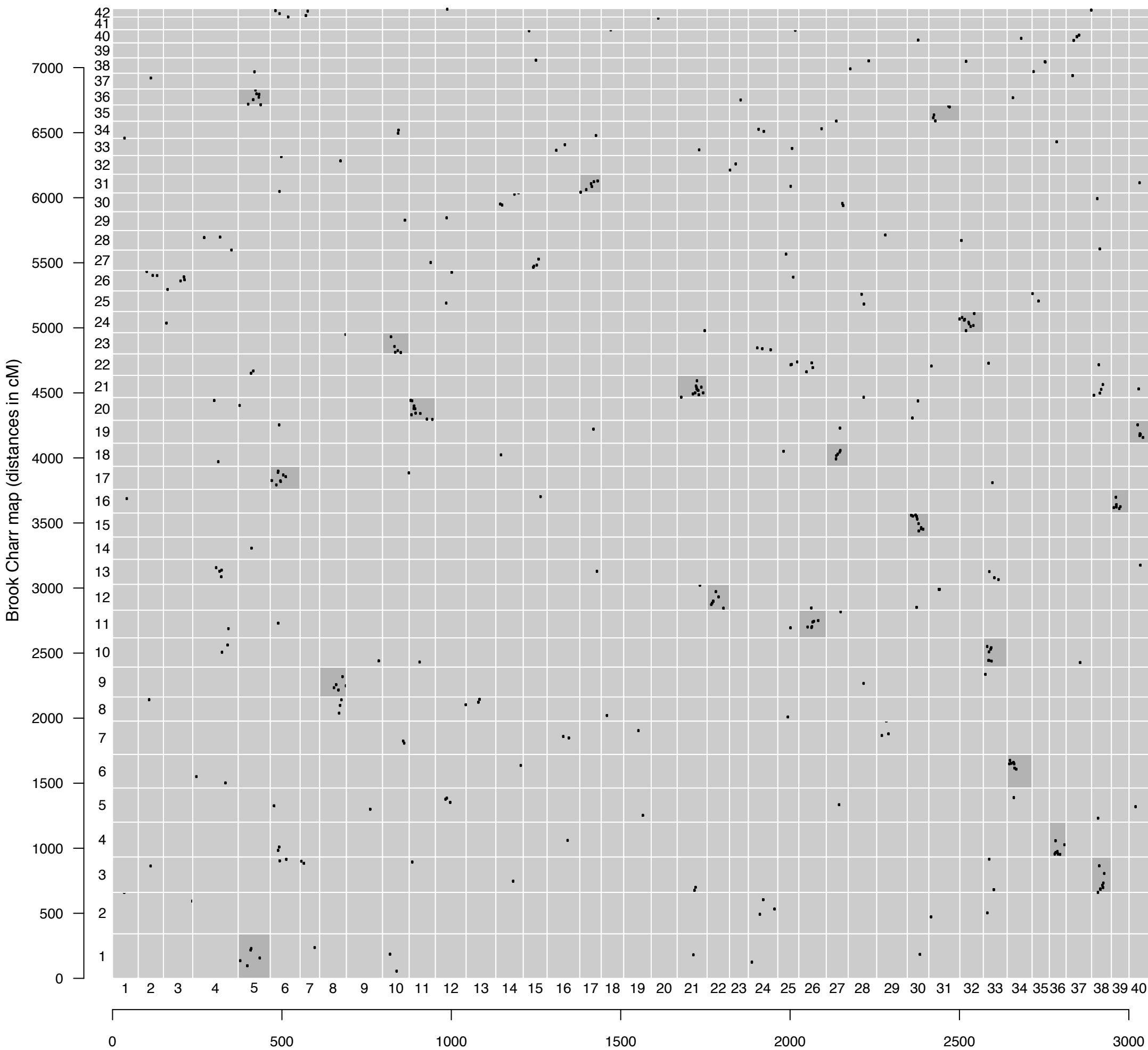

Ref. genome = RT

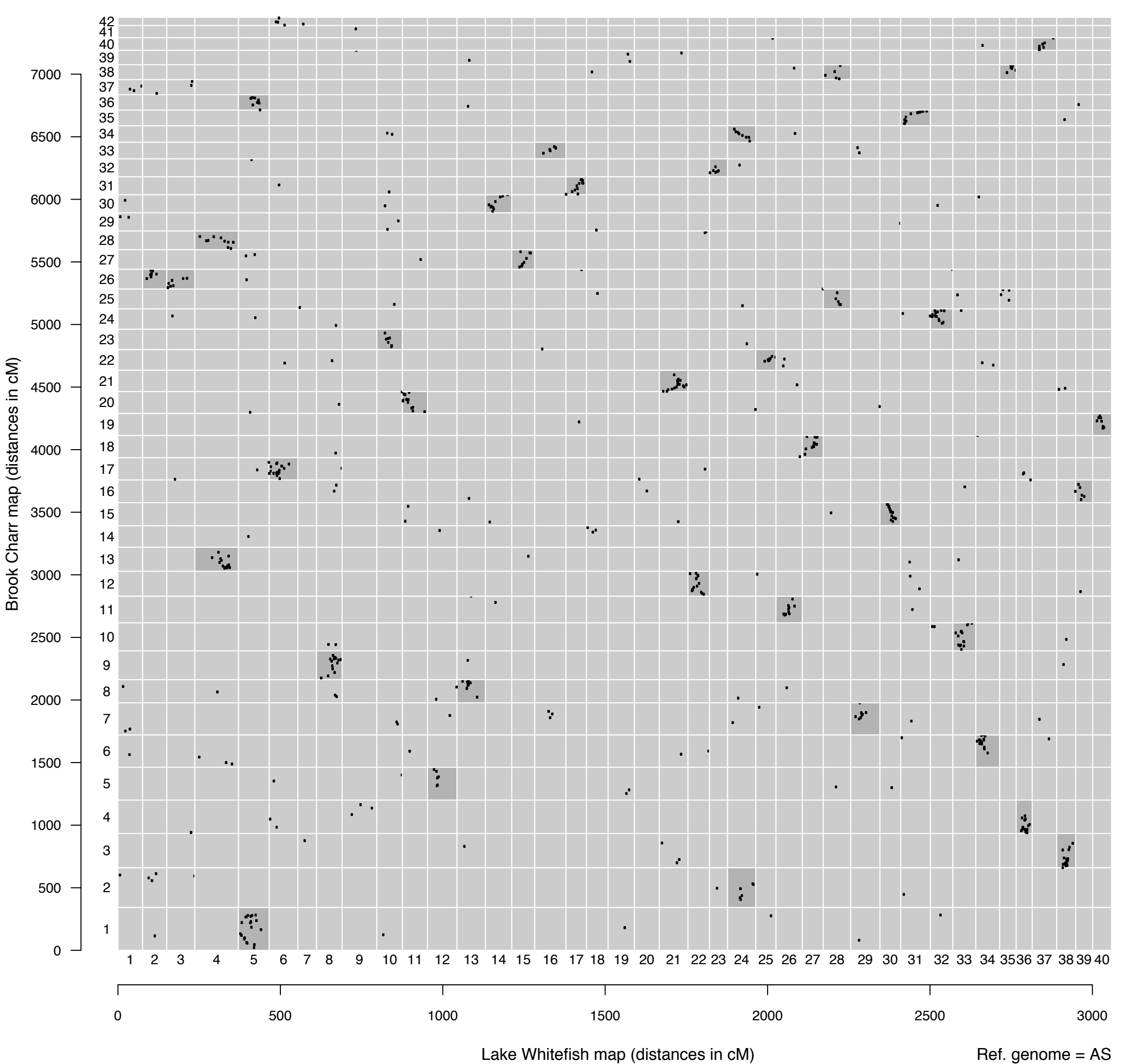

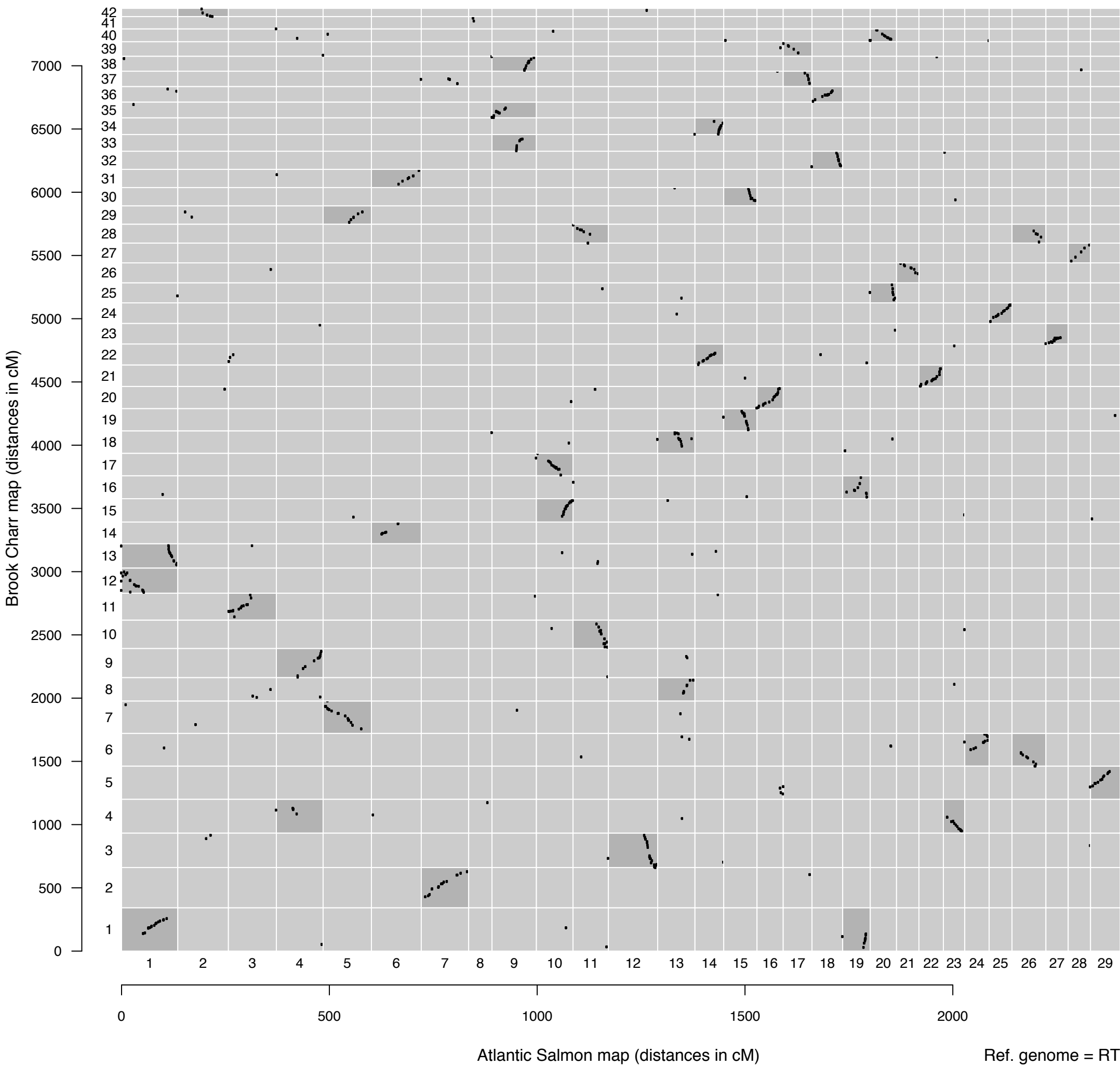

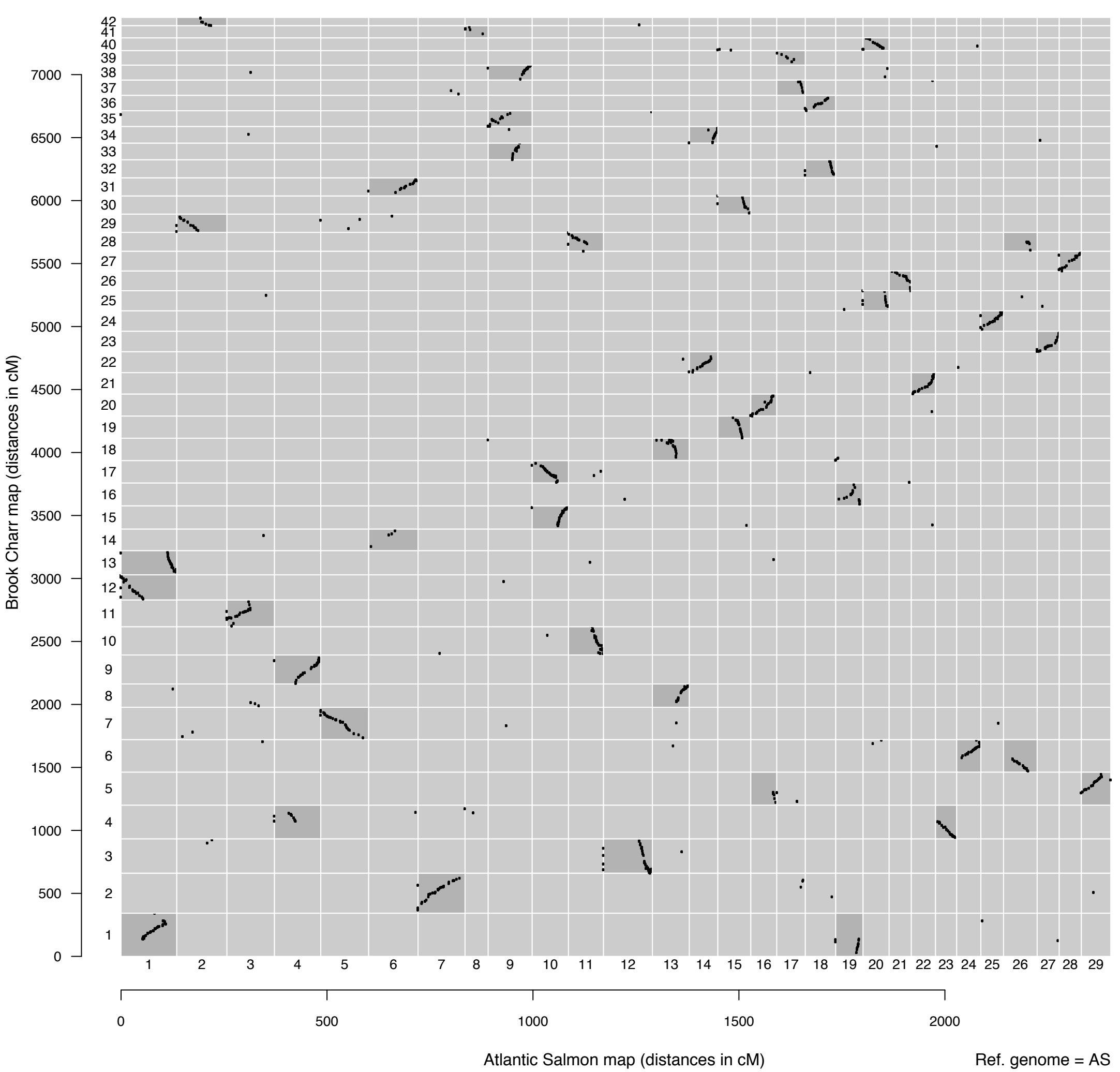

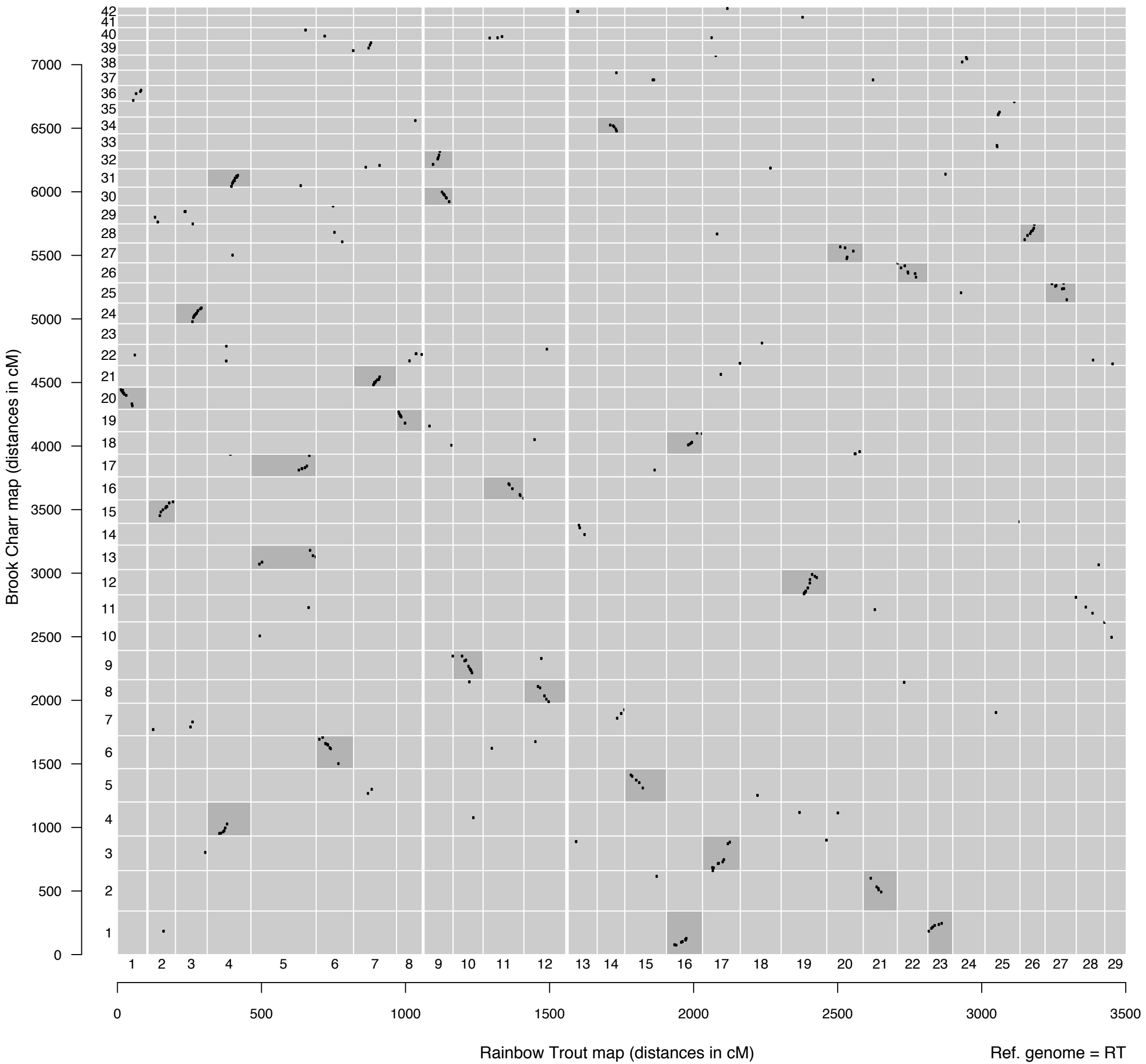

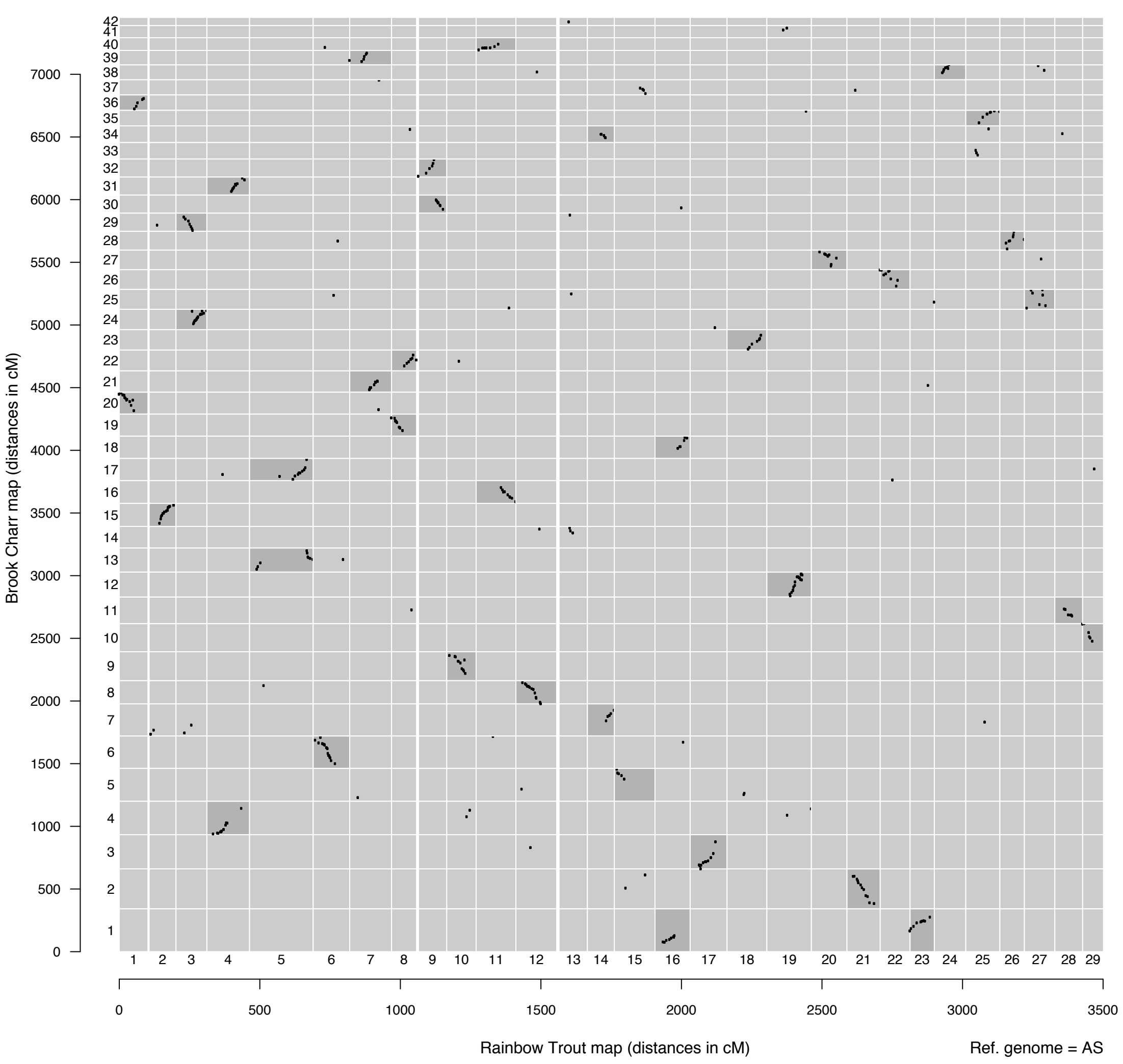

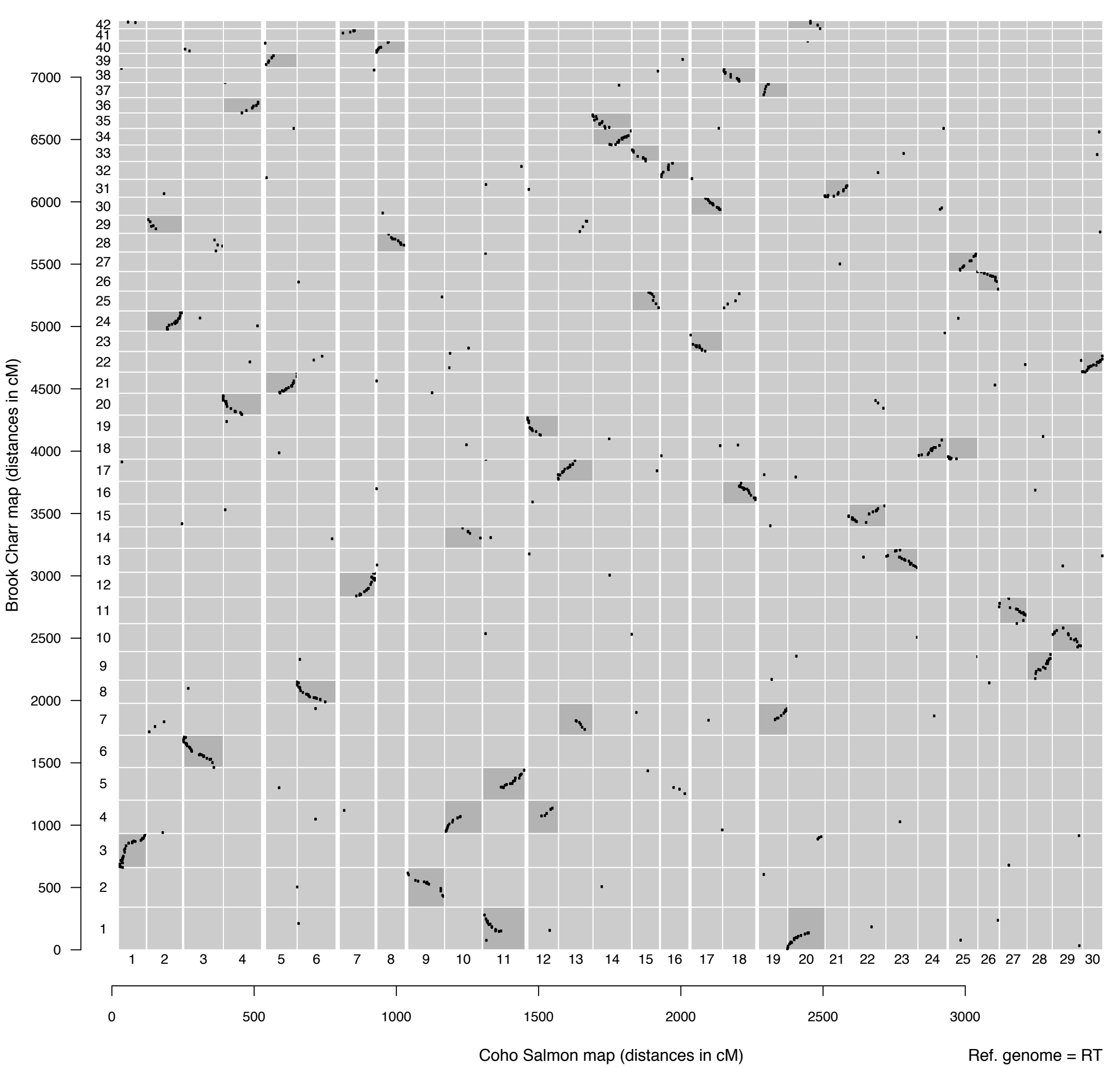

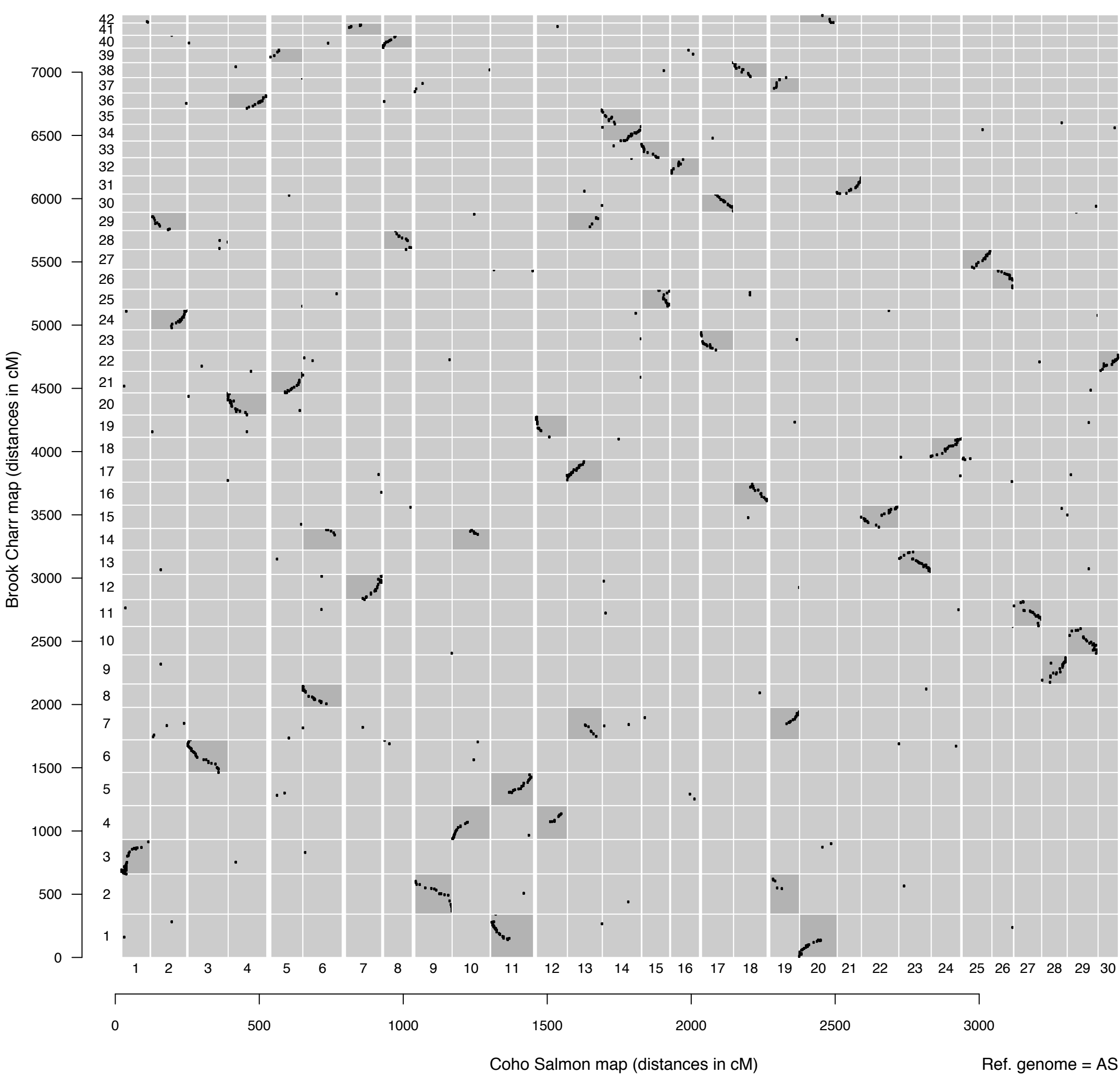

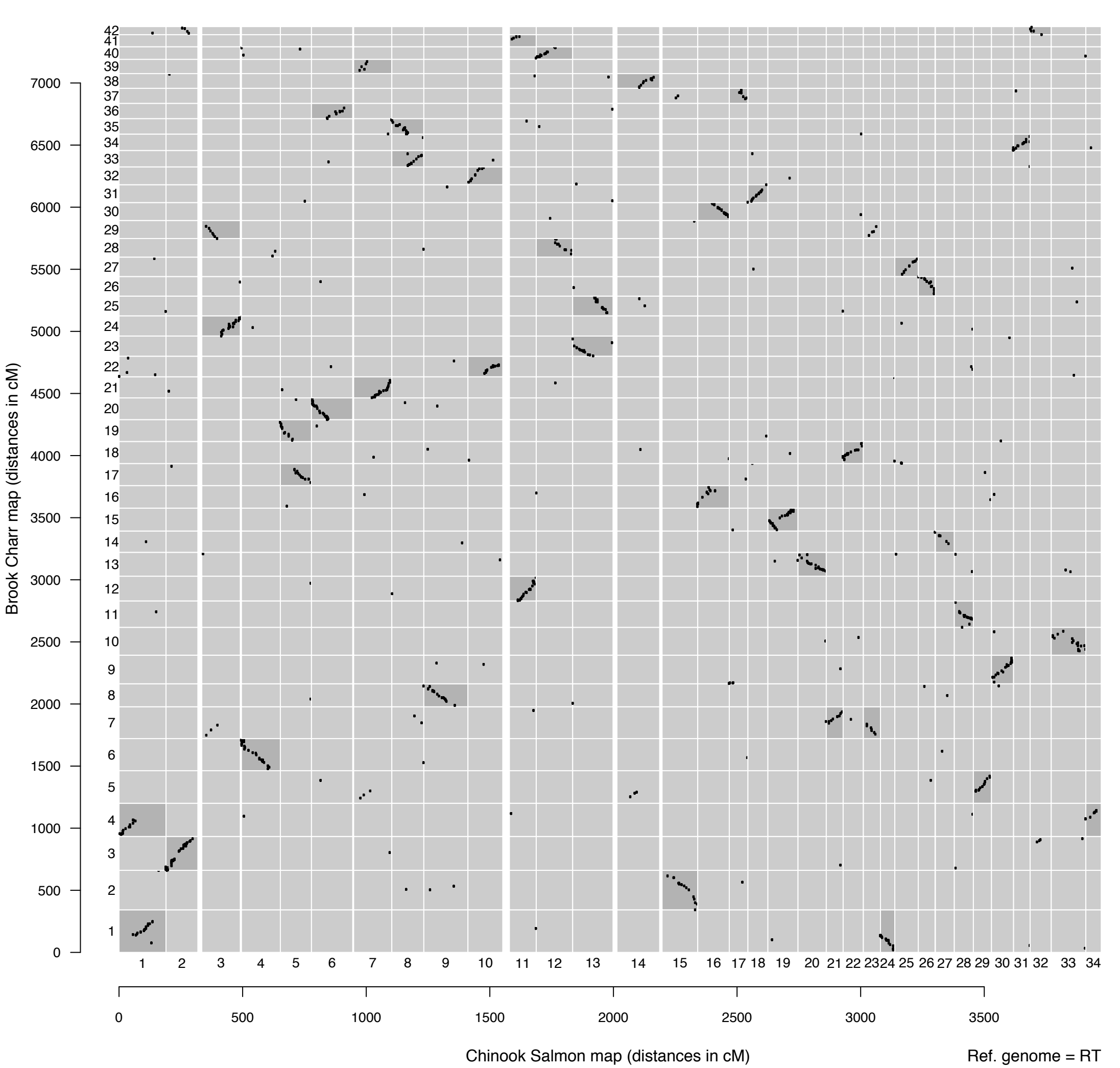

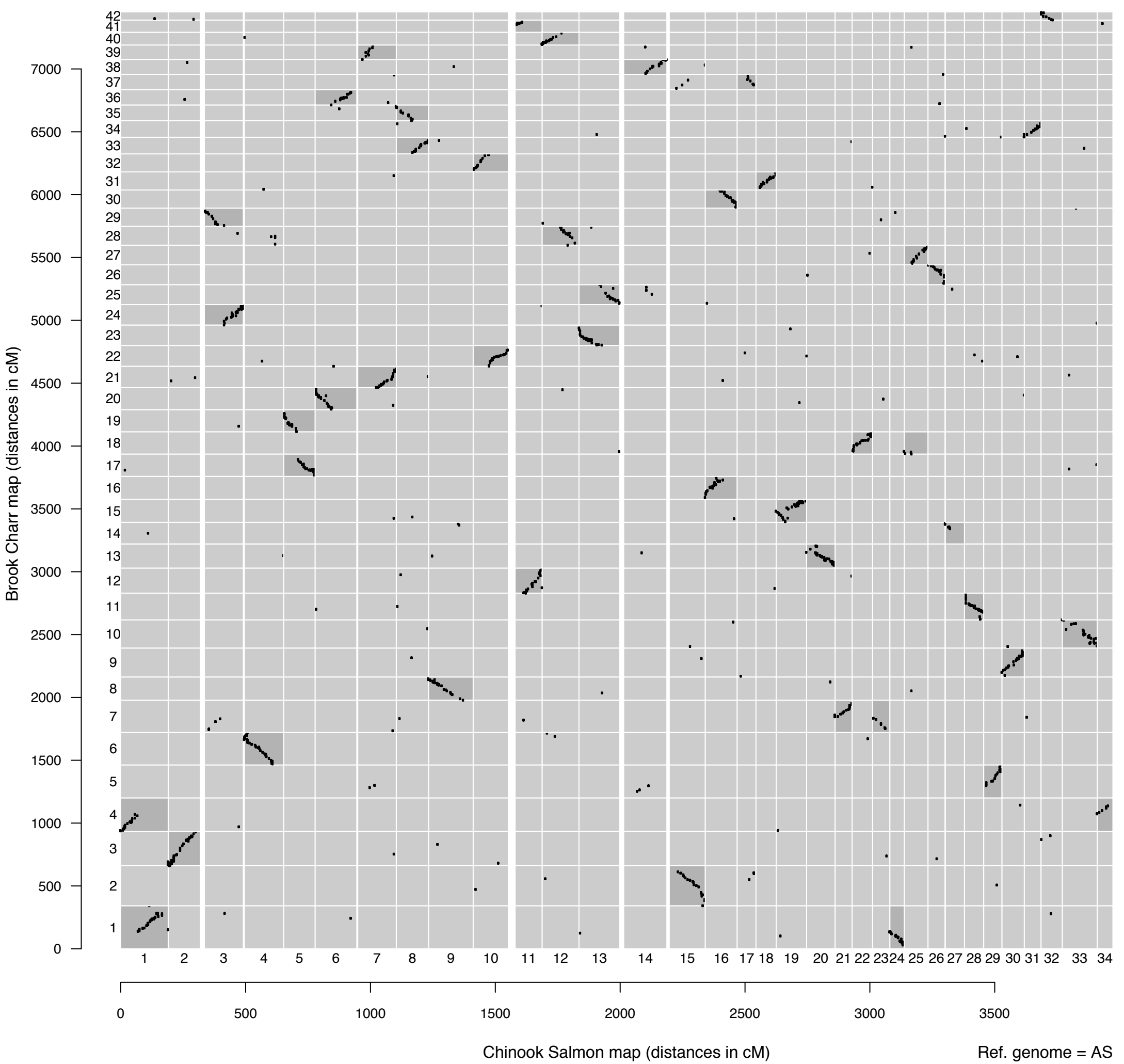

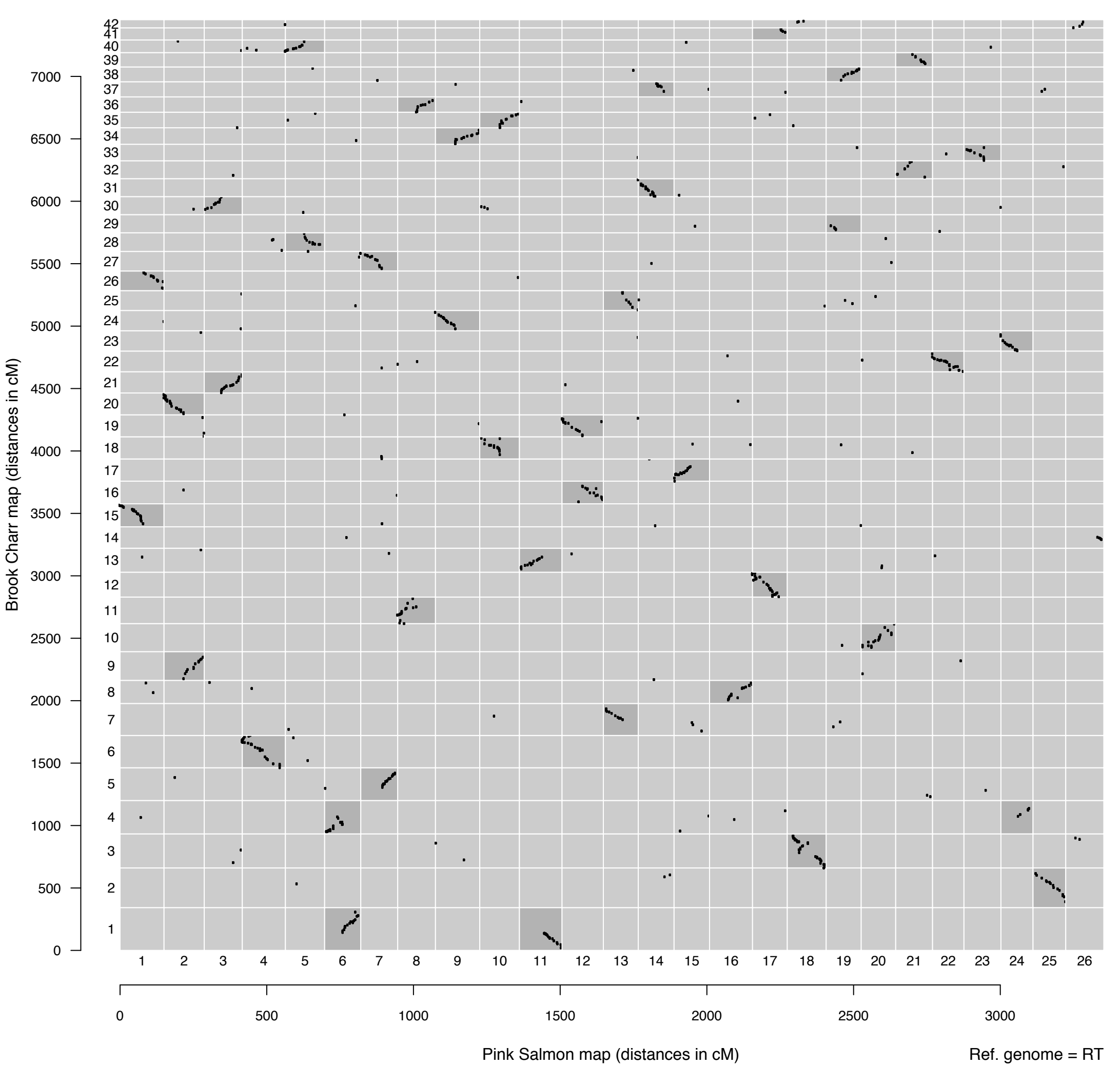

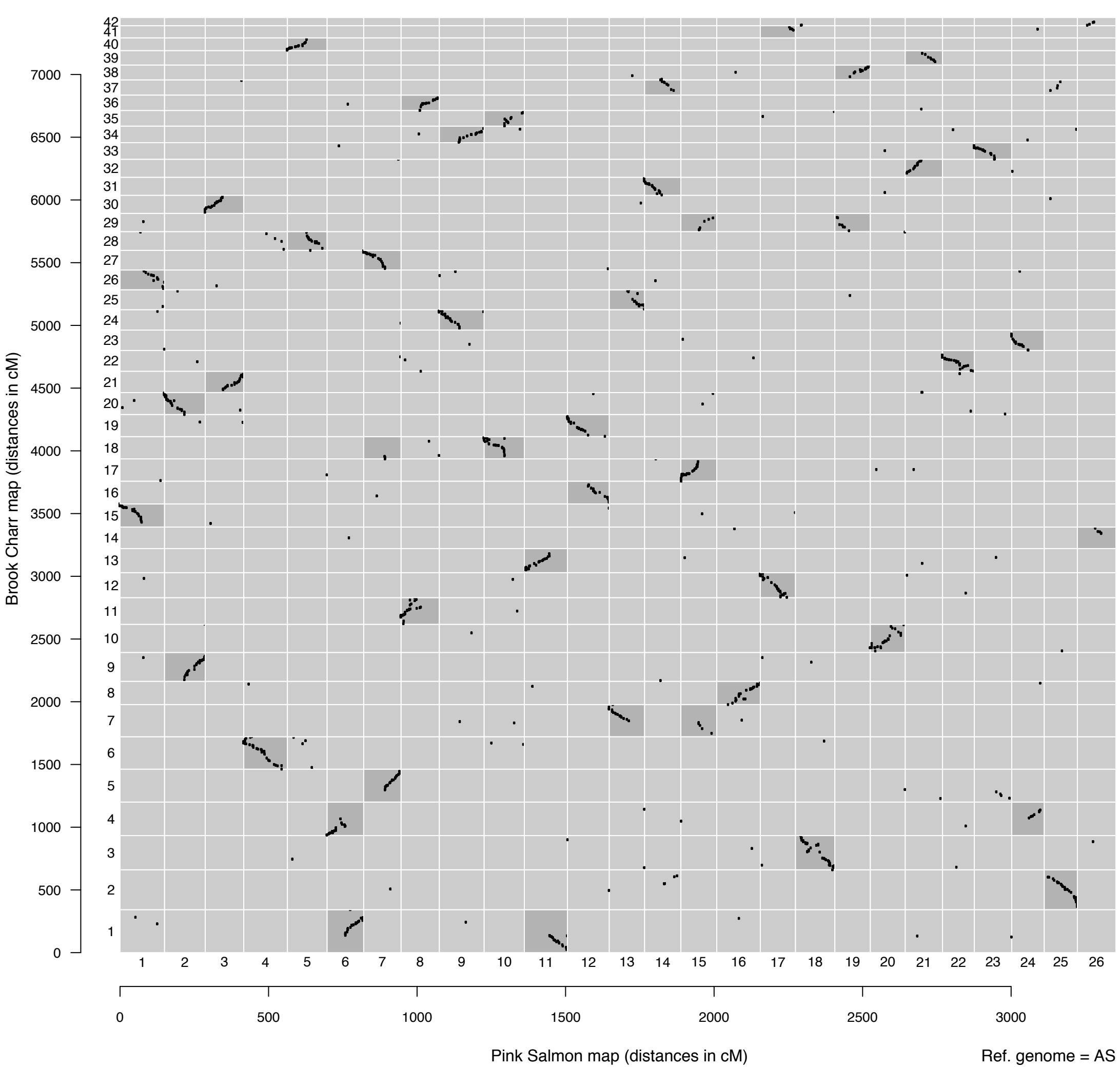

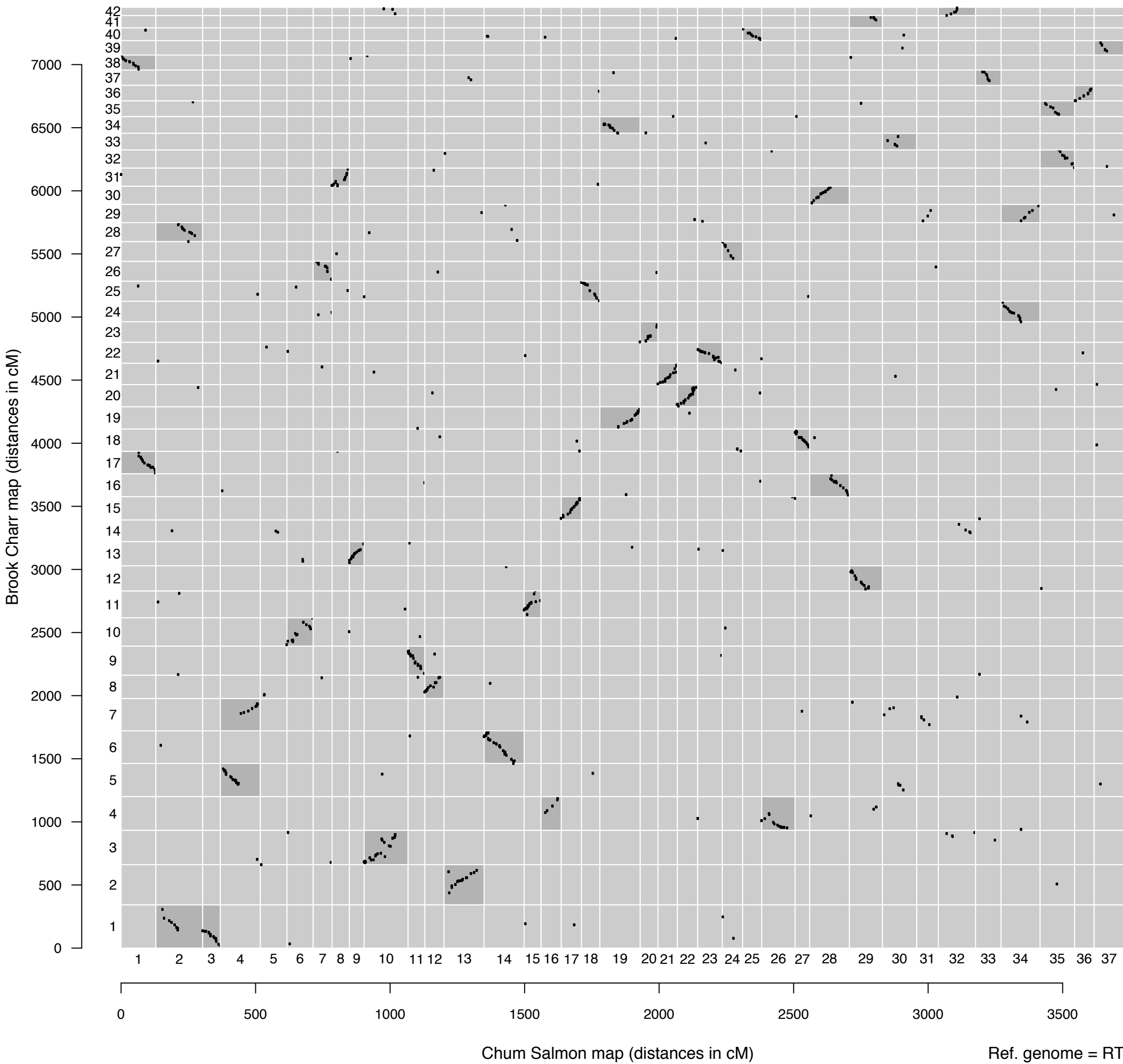

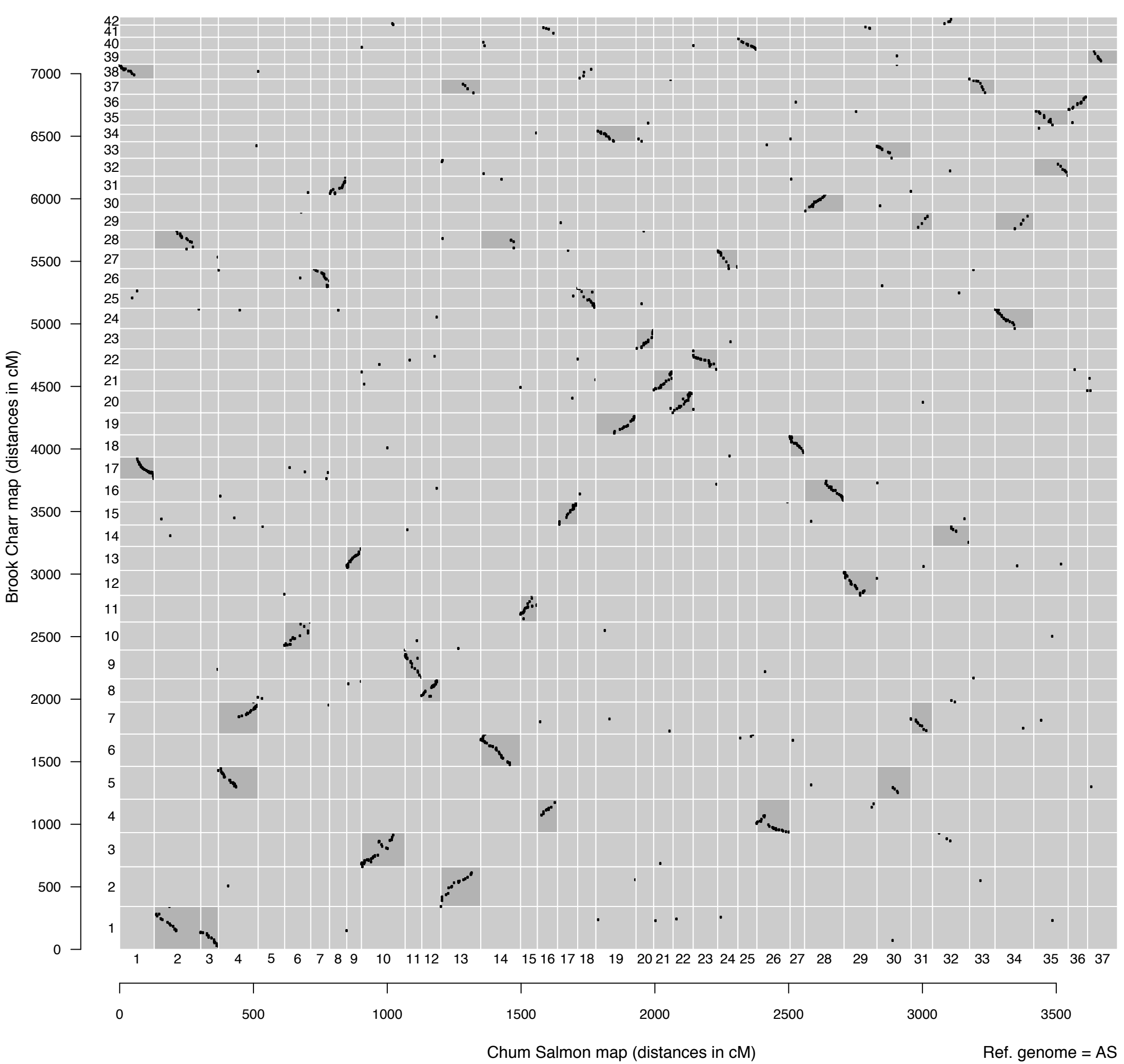

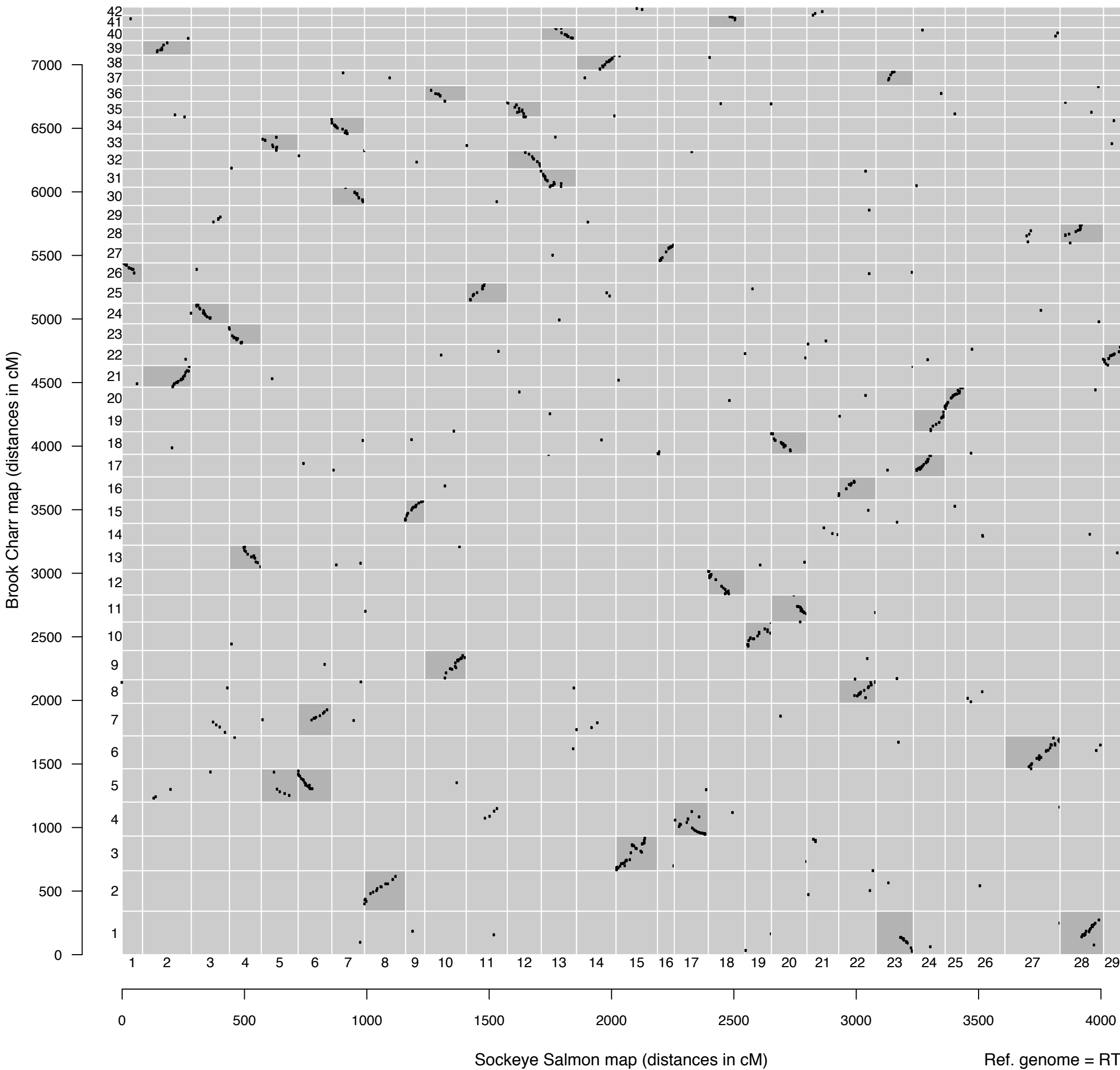

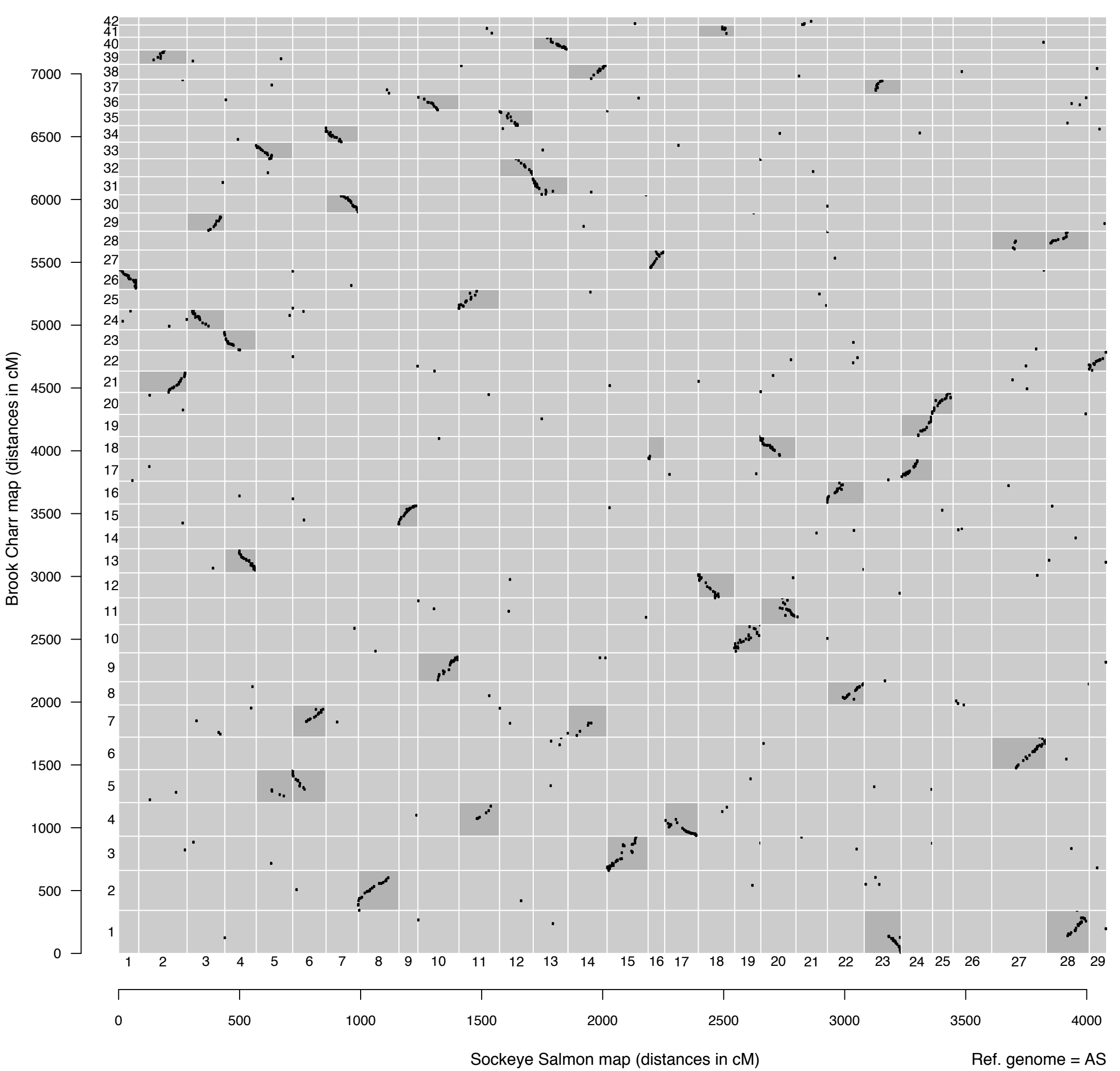

Supplement: Supplementary Data [file evw262_Supp.zip › additional_fileS5_sfon_vs_all_both_RT_and_AS_2016-09-02.pdf]
